# Supplementary material for: The RelA hydrolase domain acts as a molecular switch for (p)ppGpp synthesis
Source: Commun Biol. 2021 Mar 31;4:434. doi: 10.1038/s42003-021-01963-z (PMC8012599; doi:10.1038/s42003-021-01963-z)
Supplement: Supplementary file 2 — Supplementary Information [file 42003_2021_1963_MOESM2_ESM.pdf]

Supplementary Information

The RelA hydrolase domain acts as a molecular switch for (p)ppGpp synthesis

Anurag Kumar Sinha[1,2] and Kristoffer Skovbo Winther[1*]

[1]Department of Biology, University of Copenhagen, Ole Maaløesvej 5, DK-2200 Copenhagen, Denmark

[2]Current address: National Food Institute, Technical University of Denmark, DK-2800 Kgs. Lyngby, Denmark

*Correspondence: kristoffer.winther@bio.ku.dk

Supplementary Methods

Strains Constructed

Oligonucleotides, strains and plasmids used for strain construction are shown in Supplementary Table 1

MG1655 *relA*^{QUAD} and MG1655 *relA*^{QUAD}*::HTF*

A chloramphenicol cassette was inserted into isoleucine 116 of *relA* in MG1655 and MG1655 *relA::HTF*. The cassette was generated by PCR using plasmid pWRG100 as template and oligos I116-CM-f and I116-CM-rv and was electroporated (app. 200 ng) into MG1655 and MG1655 *relA::HTF* expressing lambda red recombinase from plasmid pWRG99[1]. After 1 h of phenotypic expression cells and were plated onto LB agar plates containing 10μg/mL chloramphenicol and 100 μg/mL ampicillin and grown at 30ºC. Insertion of cassette generated MG1655 *relA*$^{I116::cm}$ and MG1655 *relA*$^{I116::cm}$*::HTF,* which were confirmed by PCR. Alanine substitutions in R117, Q118, K120 and H123 (referred to as QUAD) were introduced by replacing the cassette in MG1655 *relA*$^{I116::cm}$ and MG1655 *relA*$^{I116::CM}$*::HTF* with a double stranded DNA oligonucleotides containing substitution mutations. Double stranded DNA was generated by hybridization of 500 pmol of quad-scarless-f and quad-scarless-rv oligonucleotides in a reaction containing 10 mM Tris-HCl pH 7.5, 100 mM NaCl and 1 mM EDTA followed by a subsequent desalting step using a G25 Spin column (GE healthcare). 25 pmol of double stranded DNA oligonucleotide was electroporated into MG1655 *relA*$^{I116::cm}$ and MG1655 *relA*$^{I116::cm}$*::HTF* expressing lambda recombinase from pWRG99 and after 1 h of phenotypic expression serially diluted and plated on LB-plates containing 100 μg/mL ampicillin and 1 μg/mL anhydrotetracycline. Positive Sce-I resistant clones were re-streaked and sequenced.

MG1655 *relA*$^{\Delta 116\text{-}129}$ and MG1655 *relA*$^{\Delta 116\text{-}129}$*::HTF*

Deletions of residues 116-129 in relA (referred to as Δ116-129) were constructed as above using hybridized oligos Delta116-129-scarless-f and Delta116-129-scarless-rv.

MG1655 *relA*^{I116L} and MG1655 *relA*^{I116L}*::HTF*

Isoleucine in position 116 was substituted for leucine (referred to as I116L and was previously described by Montero et al.[2]) as above using hybridized oligos I116L-scarless-f and I116L-scarless-rv.

MG1655 *relA*^{A121E} and MG1655 *relA*^{A121E}*::HTF*

Alanine in position 121 was substituted for glutamate (referred to as A121E) as described above using oligos A121E-scarless-f and A121E-scarless-rv.

MG1655 *relA*^{L119M} and MG1655 *relA*^{L119M}*::HTF*

Leucine 119 of RelA was substituted for methionine (referred to as L119M) as described above using hybridized oligos L119M-scarless-f and L119M-scarless-rv

MG1655 *relA*^{M113K} and MG1655 *relA*^{M113K}*::HTF*

Methionine 113 was substituted for lysine (referred to as M113K) as described above using hybridized oligos M113K-scarless-f and M113K-scarless-rv

MG1655 *relA*^{Q118L} and MG1655 *relA*^{Q118L}*::HTF*

Glutamine 118 was substituted for leucine (referred to as Q118L) as described above using oligos Q118L-scarless-f and Q118L-scarless-rv.

MG1655 *relA^{H108AR111A}::HTF*

Histidine 108 and arginine 111 was substituted for alanine (referred to as H108AR111A) in MG1655 *relA::HTF* as described above using oligos H108AR111A-scarless-f and H108AR111A –scarless-rv.

MG1655 *relA^{W39A}::HTF*

A chloramphenicol cassette was inserted into tryptophan W39 of relA in MG1655 *relA::HTF*. The cassette was generated by PCR using plasmid pWRG100 as template and oligos W39-CM-f and W39-CM-rv and was electroporated MG1655 *relA::HTF* as described previously. W39 was substituted for alanine (referred to as W39A) by replacing the cassette in MG1655 *relA$^{W39::cm}$::HTF* as described above using oligos W39A-scarless-f and W39A-scarless-rv.

MG1655 *relA$^{\Delta W39}$::HTF*

Deletion of W39 (referred to as ΔW39) was introduced as described above using oligos DeltaW39-scarless-f and DeltaW39-scarless-rv.

MG1655 *relA^{R96AK101A}::HTF*

A chloramphenicol cassette was inserted into serine S98 of relA in MG1655 *relA::HTF* as described above using a PCR product generated from oligos S98-CM-f and S98-CM-rv. R96 and K101 were substituted for alanine (referred to as R96AK101A) by replacing the cassette in MG1655 *relA$^{S98::cm}$::HTF* with hybridized oligos R96AK101A-scarless-f and R96AK101A-scarless-rv as described previously.

MG1655 *relA^{R136AR137A}::HTF*

A chloramphenicol cassette was inserted into arginine R136 of relA in MG1655 *relA::HTF* as described previously using a PCR product generated by oligos R136-CM-f and R136-

CM-rv R136 and R137 were substituted for alanine (referred to as R136AR137A) by replacing the cassette in MG1655 *relA*$^{R136::cm}$*::HTF* with a hybridized oligos R136AR137A-scarless-f and R136AR137A-scarless-rv as described above.

Protein stability by western blotting analysis

Cultures of MG1655 *ΔrelA*, MG1655 *relA::HTF*, MG1655 *relA*^{A121E}*::HTF*, MG1655 *relA*^{QUAD}*::HTF*, MG1655 *relA*^{I116L}*::HTF* and MG1655 *relA*$^{Δ116-129}$*::HTF* were grown exponentially in MOPS minimal medium supplemented with 0.2% glucose and all nucleobases (10 µg ml^{-1} of each) at 30ºC. At OD_{600} = 0.2 Isoleucine starvation was induced by addition of L-Valine to a final concentration of 500 µg/mL. A 1 mL sample was collected before and after 30 min of Isoleucine starvation and the cells pelleted by centrifugation at 4ºC. The pellet was resuspend in 50 µL 1x LDS loading buffer (Life technologies) with 5 mM DTT and boiled for 5 min. After a brief step of centrifugation at 14 krpm, 20 µL of sample was loaded onto a 4-12% NuPAGE gel (Life technologies) and the protein separated by electrophoresis in 1xMOPS running buffer (Life technologies). The protein was transferred to a PVDF membrane (Amersham) and six Histidine tagged RelA protein was detected by incubation with Penta-His primary antibodies (Qiagen) followed by incubation with HRP conjugated mouse IgG secondary antibodies (Sigma) in PBS with 0.1% Tween-20 and 5% Milk powder. The protein bands were visualized using Pierce ECL chemiluminiscense substrate (Thermo scientific) according to manufacturer's instructions and the signal detected in an Imagequant LAS4100.

Supplementary Table 1

Oligonucleotides	
I116-CM-f	GGTCGTTAACCTTATTCACGGCGTGCGTGATATGGCGGCGCTAGACTATATTACCCTGTT
I116-CM-rv	CGGAGGAAACAGAATCAGTGTGCGTCGCTTTCAGCTGGCGCGCCTTACGCCCCGCCCTGC
Quad_scarless-f	TAACCTTATTCACGGCGTGCGTGATATGGCGGCGATCGCGGCCCTGGCGGCGACGGCCACTGATTCTGTTTCCTCCGAACAGGTCGATAACG
Quad_scarless-rv	CGTTATCGACCTGTTCGGAGGAAACAGAATCAGTGGCCGTCGCCGCCAGGGCCGCGATCGCCGCCATATCACGCACGCCGTGAATAAGGTTA
Delta116-129_scarless-f	GGTCGTTAACCTTATTCACGGCGTGCGTGATATGGCGGCGGAACAGGTCGATAACGTTCGCCGGATGTTATTGGCGATGG
Delta116-129_scarless-rv	CCATCGCCAATAACATCCGGCGAACGTTATCGACCTGTTCCGCCGCCATATCACGCACGCCGTGAATAAGGTTAACGACC
I116L-scarless-f	GTTAACCTTATTCACGGCGTGCGTGATATGGCGGCGCTGCGCCAGCTGAAAGCGACGCACACTGATTCTGTTTCCTCCGA
I116L-scarless-rv	TCGGAGGAAACAGAATCAGTGTGCGTCGCTTTCAGCTGGCGCAGCGCCGCCATATCACGCACGCCGTGAATAAGGTTAAC
A121E-scarless-f	TTAACCTTATTCACGGCGTGCGTGATATGGCGGCGATCCGCCAGCTGAAAGAAACGCACACTGATTCTGTTTCCTCCGAACAGGTCGA
A121E-scarless-rv	TCGACCTGTTCGGAGGAAACAGAATCAGTGTGCGTTTCTTTCAGCTGGCGGATCGCCGCCATATCACGCACGCCGTGAATAAGGTTAA
L119M-scarless-f	TTAACCTTATTCACGGCGTGCGTGATATGGCGGCGATCCGCCAGATGAAAGCGACGCACACTGATTCTGTTTCCTCCGAACA

L119M-scarless-rv	TGTTCGGAGGAAACAGAATCAGTGTGCGTCGCTTTCATCTGGCGGATCGCCGCCATATCACGCACGCCGTGAATAAGGTTAA
M113K-scarless-f	GTCGGTCGTTAACCTTATTCACGGCGTGCGTGATAAAGCGGCGATCCGCCAGCTGAAAGCGACGCACACTGATTCTGTTTC
M113K-scarless-rv	GAAACAGAATCAGTGTGCGTCGCTTTCAGCTGGCGGATCGCCGCTTTATCACGCACGCCGTGAATAAGGTTAACGACCGAC
Q118L-scarless-f	AACCTTATTCACGGCGTGCGTGATATGGCGGCGATCCGCCTGCTGAAAGCGACGCACACTGATTCTGTTTCCTCCG
Q118L-scarless-rv	CGGAGGAAACAGAATCAGTGTGCGTCGCTTTCAGCAGGCGGATCGCCGCCATATCACGCACGCCGTGAATAAGGTT
H108AR111A-scarless-f	GTGAGAGCGTCGGTAAGTCGGTCGTTAACCTTATTGCGGGCGTGGCGGATATGGCGGCGATCCGCCAGCTGAAAGCGACGCACACTGATTCTG
H108AR111A-scarless-rv	CAGAATCAGTGTGCGTCGCTTTCAGCTGGCGGATCGCCGCCATATCCGCCACGCCCGCAATAAGGTTAACGACCGACTTACCGACGCTCTCAC
W39-CM-f	CCAGCCAGAAGTCGTGTGAGTGCTTAGCCGAAACCCTAGACTATATTACCCTGTT
W39-CM-rv	CATCCGGATGCCCCTGCGTCTGTTGCAGACAATACGCCGCCTTACGCCCCGCCCTGC
W39A-scarless-f	GTATTACCAGCCAGAAGTCGTGTGAGTGCTTAGCCGAAACCGCCGCGTATTGTCTGCAACAGACGCAGGGGCATCCGGATG
W39A-scarless-rv	CATCCGGATGCCCCTGCGTCTGTTGCAGACAATACGCGGCGGTTTCGGCTAAGCACTCACACGACTTCTGGCTGGTAATAC
DeltaW39-scarless-f	CCAGCCAGAAGTCGTGTGAGTGCTTAGCCGAAACCGCGTATTGTCTGCAACAGACGCAGGGGCATCCGGA
DeltaW39-scarless-f	TCCGGATGCCCCTGCGTCTGTTGCAGACAATACGCGGTTTCGGCTAAGCACTCACACGACTTCTGGCTGG
S98-CM-f	GATGCCAACGTAGTCAGCGAAGATGTGCTGCGTGAGCTAGACTATATTACCCTGTT

S98-CM-rv	CACGCCGTGAATAAGGTTAACGACCGACTTACCGACCGCCTTACGCCCCGCCCT GC
R96AK101A-scarless-f	CTGGCGGATGCCAACGTAGTCAGCGAAGATGTGCTGGCGGAGAGCGTCGGTGC GTCGGTCGTTAACCTTATTCACGGCGTGCGTGATATG
R96AK101A-scarless-rv	CATATCACGCACGCCGTGAATAAGGTTAACGACCGACGCACCGACGCTCTCCGC CAGCACATCTTCGCTGACTACGTTGGCATCCGCCAG
R136-CM-f	CTGATTCTGTTTCCTCCGAACAGGTCGATAACGTTCTAGACTATATTACCCTGTT
R136-CM-rv	CAGCGAAAATCATCGACCATCGCCAATAACATCCGCGCCTTACGCCCCGCCCTGC
R136AR137A-scarless-f	CTGATTCTGTTTCCTCCGAACAGGTCGATAACGTTGCGGCCATGTTATTGGCGAT GGTCGATGATTTTCGCTGCG
R136AR137A-scarless-rv	CGCAGCGAAAATCATCGACCATCGCCAATAACATGGCCGCAACGTTATCGACCTG TTCGGAGGAAACAGAATCAG
Loop-mut-f	CGGTCGTTAACCTTATTCAC
Loop-mut-rv	CTACGCAGCGAAAATCATC
miRCat-33	rAppTGGAATTCTCGGGTGCCAAGG/ddC/
33-rev	CCTTGGCACCCGAGAATT
L5Aa	InvddT/ACACrGrArCrGrCrUrCrUrUrCrCrGrArUrCrUrNrNrNrUrArArGrC
L5Ab	InvddT/ACACrGrArCrGrCrUrCrUrUrCrCrGrArUrCrUrNrNrNrArUrUrArGrC
L5Ad	InvddT/ACACrGrArCrGrCrUrCrUrUrCrCrGrArUrCrUrNrNrNrCrGrCrUrUrArGrC
L5Bb	InvddT/ACACrGrArCrGrCrUrCrUrUrCrCrGrArUrCrUrNrNrNrGrUrGrArGrC
L5Bc	InvddT/ACACrGrArCrGrCrUrCrUrUrCrCrGrArUrCrUrNrNrNrCrArCrUrArGrC
L5Bd	InvddT/ACACrGrArCrGrCrUrCrUrUrCrCrGrArUrCrUrNrNrNrUrCrUrCrUrArGrC
P5	AATGATACGGCGACCACCGAGATCTACACTCTTTCCCTACACGACGCTCTT CCGATCT
PE	CAAGCAGAAGACGGCATACGAGATCGGTCTCGGCATTCCTGGCCTTGGCA CCCGAGAATTCC
Strains	
MG1655	*Escherichia coli* K-12 wild type (Lab collection)

MG1655<br>*relA::HTF*	Winther et al.[3]
MG1655 *ΔrelA*	Sinha et al.[4]
Plasmids	
pWRG99	Blank et al.[1]
pWRG100	Blank et al.[1]

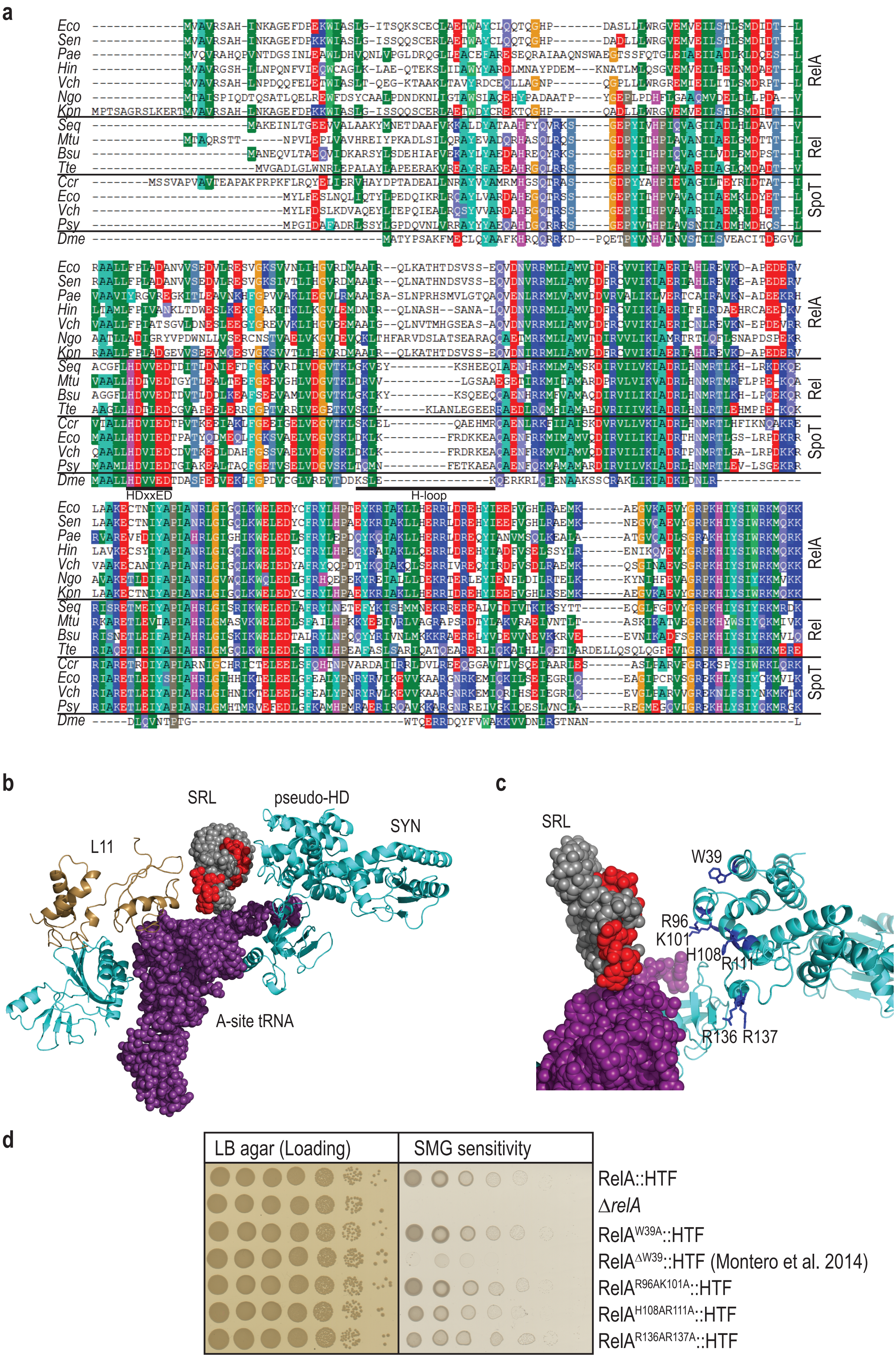

Supplementary Figure 1 - Page 1

e

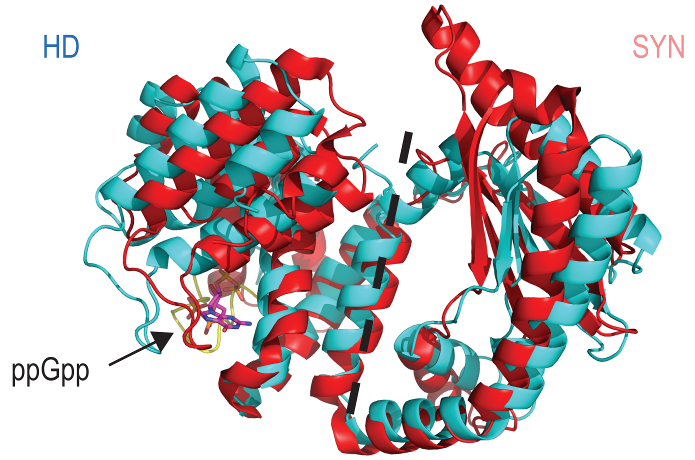

f

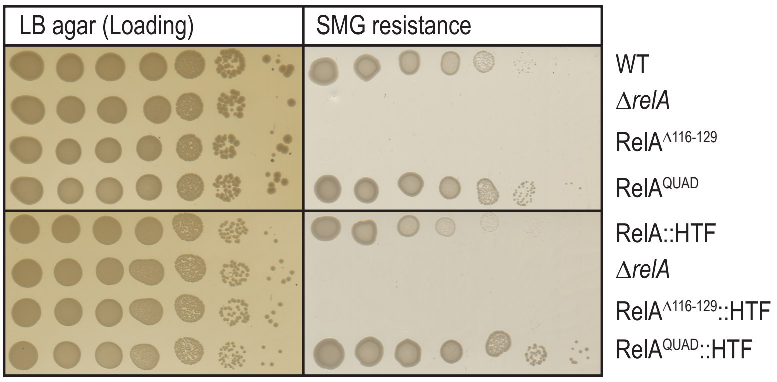

g

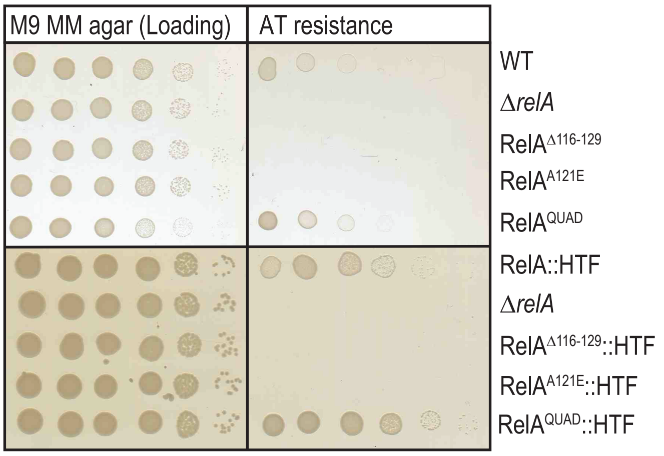

h

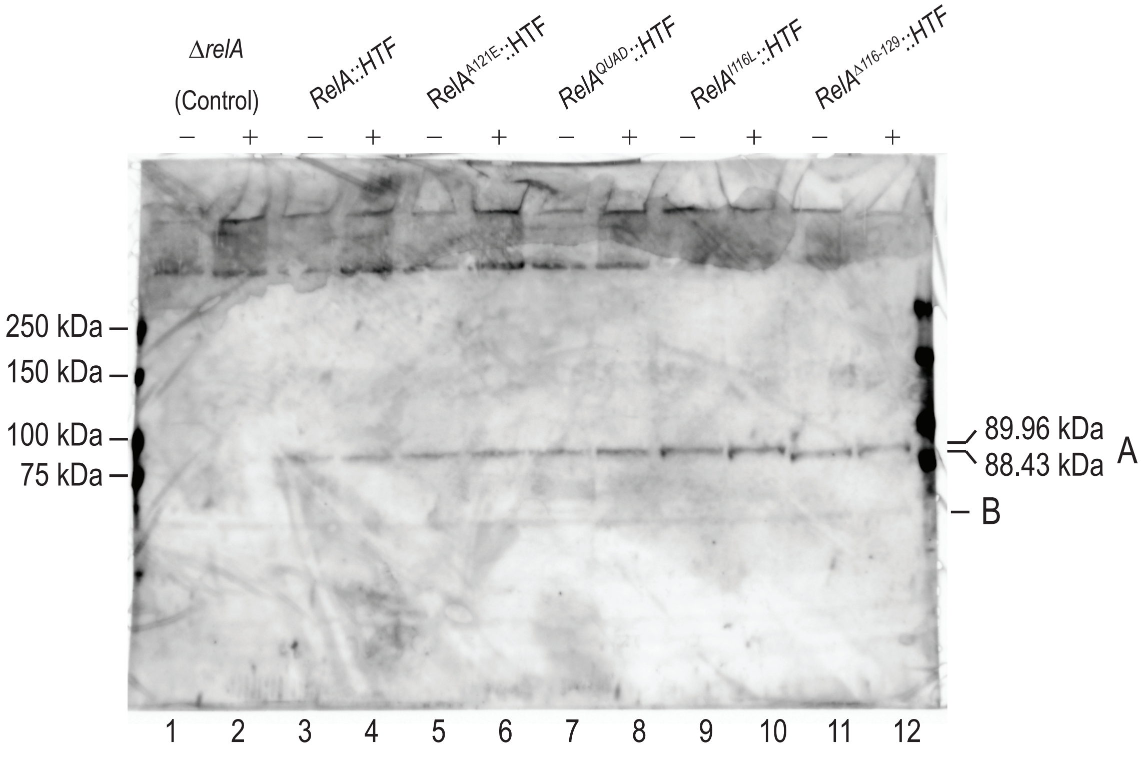

Supplementary Figure 1 - Page 2

i

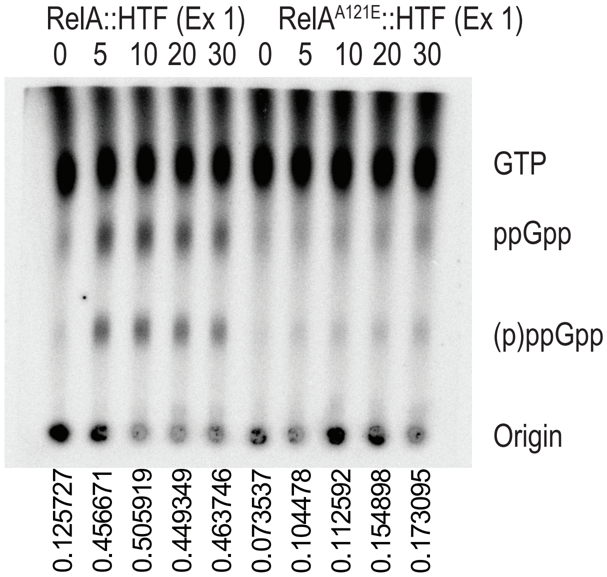
RelA::HTF (Ex 1) RelA^{A121E}::HTF (Ex 1)
0 5 10 20 30 0 5 10 20 30
GTP
ppGpp
(p)ppGpp
Origin
0.125727 0.456671 0.505919 0.449349 0.463746 0.073537 0.104478 0.112592 0.154898 0.173095

j

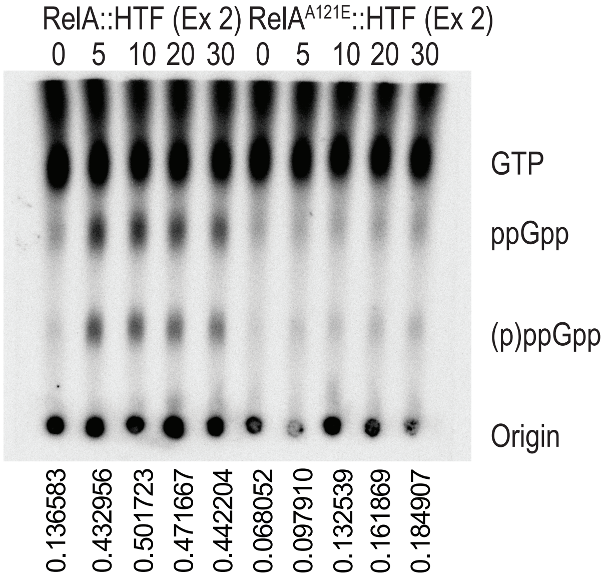
RelA::HTF (Ex 2) RelA^{A121E}::HTF (Ex 2)
0 5 10 20 30 0 5 10 20 30
GTP
ppGpp
(p)ppGpp
Origin
0.136583 0.432956 0.501723 0.471667 0.442204 0.068052 0.097910 0.132539 0.161869 0.184907

k

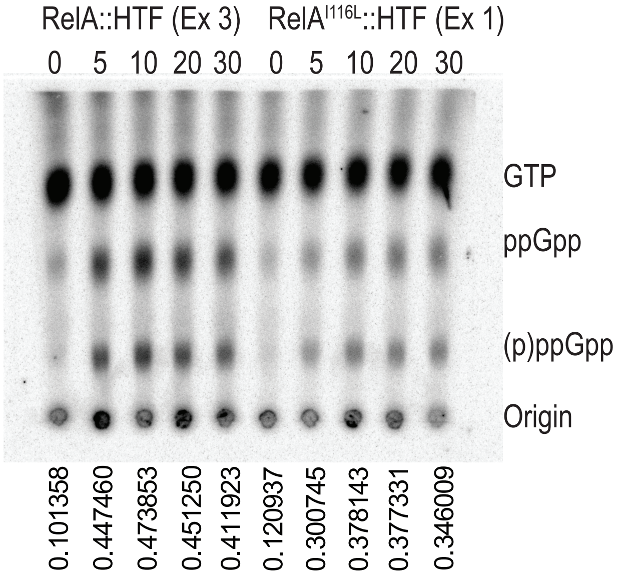
RelA::HTF (Ex 3) RelA^{I116L}::HTF (Ex 1)
0 5 10 20 30 0 5 10 20 30
GTP
ppGpp
(p)ppGpp
Origin
0.101358 0.447460 0.473853 0.451250 0.411923 0.120937 0.300745 0.378143 0.377331 0.346009

l

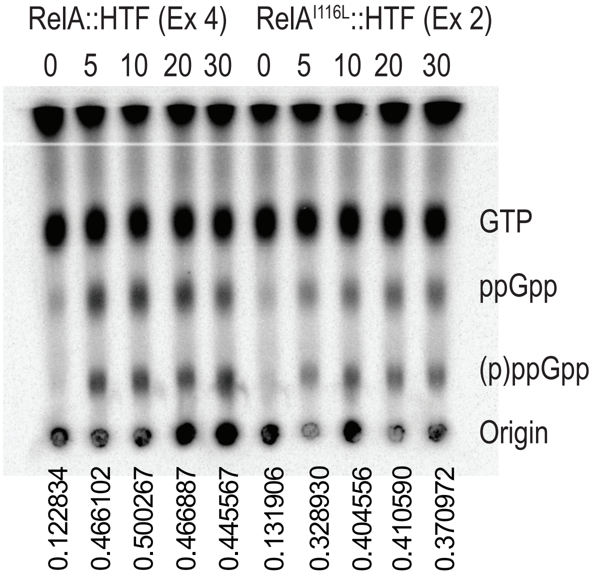
RelA::HTF (Ex 4) RelA^{I116L}::HTF (Ex 2)
0 5 10 20 30 0 5 10 20 30
GTP
ppGpp
(p)ppGpp
Origin
0.122834 0.466102 0.500267 0.466887 0.445567 0.131906 0.328930 0.404556 0.410590 0.370972

m

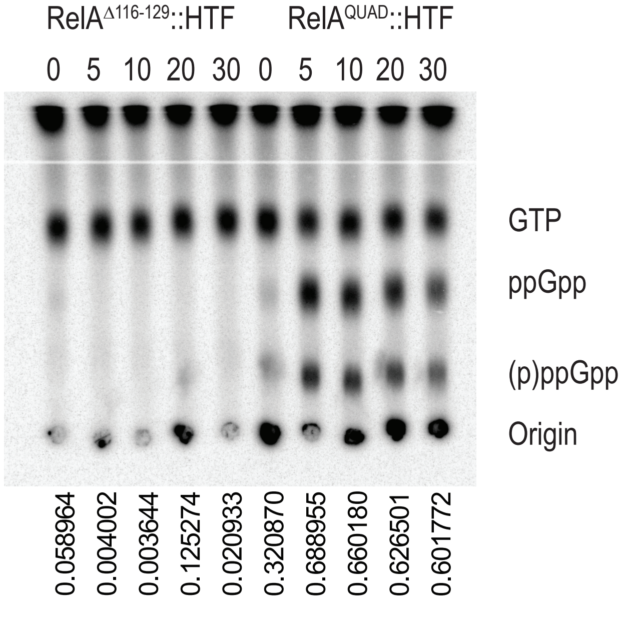
RelA$^{\Delta 116-129}$::HTF RelA^{QUAD}::HTF
0 5 10 20 30 0 5 10 20 30
GTP
ppGpp
(p)ppGpp
Origin
0.058964 0.004002 0.003644 0.125274 0.020933 0.320870 0.688955 0.660180 0.626501 0.601772

n

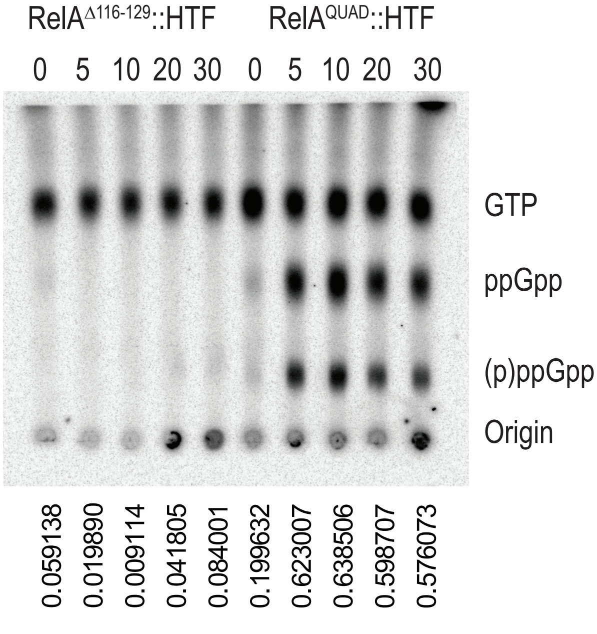
RelA$^{\Delta 116-129}$::HTF RelA^{QUAD}::HTF
0 5 10 20 30 0 5 10 20 30
GTP
ppGpp
(p)ppGpp
Origin
0.059138 0.019890 0.009114 0.041805 0.084001 0.199632 0.623007 0.638506 0.598707 0.576073

Supplementary Figure 1: Identification of the H-loop in the hydrolase domain. a Multiple sequence alignment of selected RelA, Rel and SpoT N-terminal sequences *Eco, Escherichia coli* (RelA: NP_417264.1, SpoT: NP_418107.1)*, Sen, Salmonella enterica* (NP_461877.1)*, Pae, Pseudomonas aeruginosa* (NP_249625.1)*, Hin, Hemophilus influenza* (WP_011271992.1)*, Vch, Vibrio cholera* (RelA: WP_000226858.1, SpoT: WP_010895463.1)*, Ngo, Neisseria gonorrhoeae* (AP023075.1)*, Kpn, Klebsiella pneumoniae* (CP006918.1)*, Seq, Streptococcus dysgalactiae subsp. equisimilis* (Q54089)*, Mtu, Mycobacterium tuberculosis* (NP_217099)*, Bsu, Bacillus subtilis* (NP_390638)*,Tte, Thermus thermophilus* (WP_011173739.1), *Ccr, Caulobacter crescentus* (WP_010919427.1)*, Psy, Pseudomonas syringae* (WP_003096603.1)*, Dme, Drosophila melanogaster* (NP_651682.1). Location of the H-loop is indicated with a box and conserved metal-dependent pyrophosphohydrolase HDXXED motif is underlined. **b** Structure of RelA (cyan) bound with uncharged tRNA (magenta) at the ribosomal A-site (Adapted from PDB: 5IQR). RelA forms an elongated structure on the ribosome enclosing the A-site tRNA. Potential RelA interaction sites with the Sarcin-Ricin Loop (SRL) are indicated in red and ribosomal protein L11 is shown in brown. **c** Close-up on residues in RelA pseudo-HD (blue) predicted to interact with the Sarcin-Ricin Loop (SRL). **d** Assaying functionality of RelA hydrolase substitution mutants. MG1655 *relA::HTF*, *ΔrelA*, $relA^{W39A}$*::HTF*, $relA^{\Delta W39}$*::HTF*, $relA^{R96AK101A}$*::HTF*, $relA^{H108AR111A}$*::HTF* and $relA^{R136AR137A}$*::HTF* were grown in LB medium. The cells were washed in PBS, serial diluted and spotted on loading control plates (LB agar) and MOPS MM SMG plates (SMG resistance) and grown at 30ºC. **e** Overlay of N-terminal domain (hydrolase and synthetase) of Rel_{Tte} from *Thermus thermophiles* (PDB: 6S2T shown in red) with N-terminal domain of RelA (PDB: 5IQR). Location of ppGpp in the Rel_{Tte} structure is

indicated. The H-loop in RelA is indicated in yellow and the dotted line separates the two functional domains. **f** Functionality test of tagged and untagged RelA H-loop deletion mutants. MG1655 (WT), MG1655 *ΔrelA*, MG1655 *relA*$^{Δ116\text{-}129}$ and *relA*^{QUAD} or HTF-tagged (C-terminal six histidine, TEV cleavage site and three FLAG epitopes) versions were grown overnight in LB medium at 37ºC. The cultures were then washed in PBS serial diluted and plated onto LB agar (loading) and MOPS MM SMG plates (SMG resistance). Un-tagged strains were grown at 37ºC and tagged strains at 30ºC. **g** MG1655 (WT), MG1655 *ΔrelA*, MG1655 *relA*$^{Δ116\text{-}129}$, MG1655 *relA*^{A121E} and *relA*^{QUAD} or HTF-tagged versions were grown and diluted as in f) and spotted onto M9 minimal agar plates containing all amino acids except histidine (FN19), 1 mM adenine, 1 mM thiamine without or with 15 mM 3-amino-1,2,3-triazole (AT resistance). Un-tagged strains were grown at 37ºC and tagged strains at 30ºC. **h** Detection of chromosomally encoded HTF-tagged RelA. MG1655 *ΔrelA* (Control), MG1655 *relA::HTF*, *relA*^{A121E}*::HTF*, *relA*^{QUAD}*::HTF*, *relA*^{I116L}*::HTF* and *relA*$^{Δ116\text{-}129}$*::HTF* were grown exponentially in MOPS minimal medium at 30ºC. Samples were collected before (-) and 30 min (+) isoleucine starvation. HTF tagged protein was detected by western analysis using penta-his antibodies as described in methods. A, indicates position of RelA::HTF (89.96 kDa) and RelA$^{Δ116\text{-}129}$::HTF (88.43 kDa), B indicates position of unspecific band present in all samples (including *ΔrelA*), which is used as a loading control. **i-n** Biological replicate (p)ppGpp measurements in MG1655 *relA::HTF, relA*^{A121E}*::HTF* , *relA*^{I116L}*::HTF, relA*$^{Δ116\text{-}129}$*::HTF* and *relA*^{QUAD}*::HTF*. Cells were grown exponentially in MOPS minimal medium containing ^{32}P-labeled phosphate at 30ºC. Isoleucine starvation was induced by addition of L-valine, to a final concentration of 500 μg/mL. Samples were collected before (time zero) and after starvation (in minutes), precipitated and separated by thin layer chromatography. Position

of loading origin, GTP, ppGpp and pppGpp for each experiment (Ex) is indicated. Numbers below the TLCs indicate the quantified fraction of (p)ppGpp of (p)ppGpp+GTP for each lane.

a

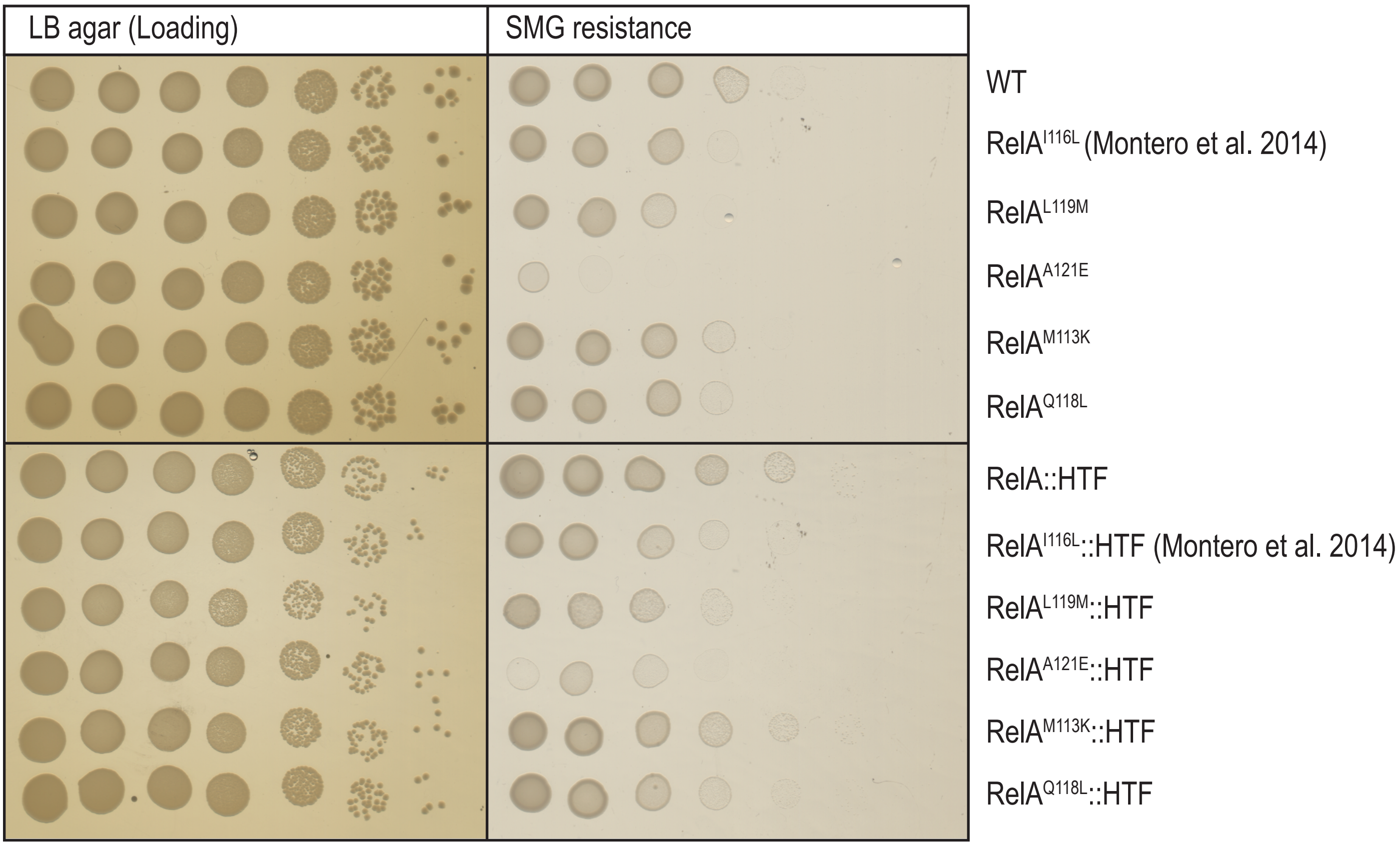

Supplementary Figure 2

Supplementary Figure 2: Substitution mutations in the H-loop modulates (p)ppGpp synthesis. a Functionality testing of untagged or tagged RelA H-loop substitution mutants. MG1655 (WT), *ΔrelA*, *relA*^{I116L}*, relA*^{L119M}, *relA*^{A121E}, *relA*^{M113K} and *relA*^{Q118L} or HTF-tagged versions were grown overnight in LB medium at 37ºC. The cultures were then washed in PBS serial diluted and plated onto LB agar (Loading) and MOPS MM SMG plates (SMG resistance). Un-tagged strains were grown at 37ºC and tagged strains at 30ºC.

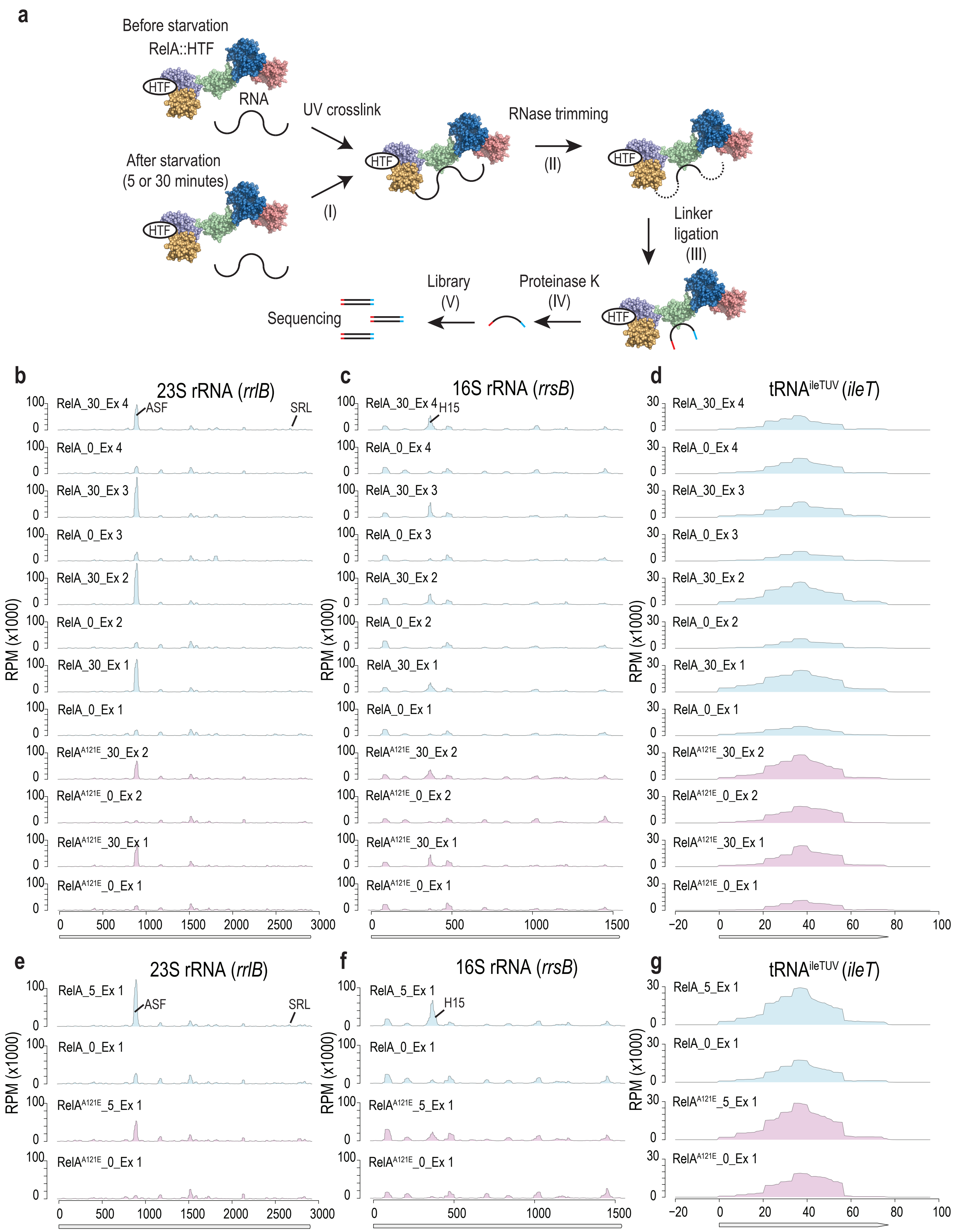
a
Before starvation
RelA::HTF
HTF
RNA
After starvation
(5 or 30 minutes)
UV crosslink
(I)
RNase trimming
(II)
Linker
ligation
(III)
Proteinase K
(IV)
Library
(V)
Sequencing
b
23S rRNA (rrlB)
ASF
SRL
c
16S rRNA (rrsB)
H15
d
tRNAileTUV (ileT)
RPM (x1000)
RelA_30_Ex 4
RelA_0_Ex 4
RelA_30_Ex 3
RelA_0_Ex 3
RelA_30_Ex 2
RelA_0_Ex 2
RelA_30_Ex 1
RelA_0_Ex 1
RelAA121E_30_Ex 2
RelAA121E_0_Ex 2
RelAA121E_30_Ex 1
RelAA121E_0_Ex 1
e
f
g
RelA_5_Ex 1
RelAA121E_5_Ex 1

h

	A-site finger (ASF) nt 834-927 in *rrlB*	Helix 15 (H15) nt 328-407 in *rrsB*	Sarcin-Ricin Loop (SRL) nt 2652-2673 in *rrlB*	$tRNA^{ileTUV}$ nt 16-56 in *ileT*
RelA_0_Ex1	25663	8121	1301	10964
RelA_0_Ex2	27485	5395	644	9546
RelA_0_Ex3	37292	10252	1139	11212
RelA_0_Ex4	32951	10538	1435	18232
RelA_0 Average	30848	8577	1130	12489
RelA_30_Ex1	139432	37873	6012	26416
RelA_30_Ex2	171142	44823	5973	27341
RelA_30_Ex3	162740	56297	2502	18401
RelA_30_Ex4	106903	56179	6090	18551
RelA_30 Average	145054	48793	5144	22677
$RelA^{A121E}$_0_Ex1	21848	6644	639	11243
$RelA^{A121E}$_0_Ex2	11768	5947	1210	19446
$RelA^{A121E}$_0 Average	16808	6295	924	15345
$RelA^{A121E}$_30_Ex1	91746	45920	1972	24603
$RelA^{A121E}$_30_Ex2	78675	41199	3517	29752
$RelA^{A121E}$_30 Average	85211	43559	2744	27178
RelA_5_Ex1	135748	71672	5360	31609
$RelA^{A121E}$_5_Ex1	61008	25370	2918	31170

Supplementary Figure 3: RelA-RNA interactions by Crosslinking and analysis of cDNAs (CRAC). a Method overview. Step (I): UV-irradiation of cell samples before and 5 or 30 minutes after isoleucine acid starvation. Step (II): Purification of RelA-RNA complexes via the FLAG epitope tag and RNA trimming by RNases. Step (III): Immobilization of complexes via the His-tag and DNA linker ligation to RNA 3'- and 5'-ends. Step (IV): Size selection of RelA-RNA complexes and RelA degradation by protease K treatment. Step (V): Finally, the cDNA libraries were generated by RT-PCR and subjected to deep sequencing. **b** DNA reads mapping to 23S rRNA (*rrlB*) obtained from CRAC analysis of MG1655 *relA::HTF and relA*^{A121E}*::HTF* before (0) and after isoleucine starvation (number indicates time in minutes). Reads are normalized to Reads per million (RPM). **c** Reads aligning to 16S rRNA (*rrsB*). **d** Reads aligning to isoleucine tRNA (*ileT*). **e** Normalized Reads obtained after short-term starvation (5 min) aligned to 23S rRNA **f** 16S rRNA or **g** isoleucine tRNA. Position of A-site finger (ASF), Sarcin-ricin Loop (SRL) and Helix 15 (H15) are indicated with arrows. Each experiment (Ex) refers to an independent biological replicate. **h** Normalized cDNA coverage (Reads per million, RPM) at RelA binding sites for samples shown in b-g). Binding sites in the ribosome (A-site finger and Sarcin-Ricin Loop of 23S rRNA encoded by *rrlB* gene and Helix 15 of 16S rRNA encoded by *rrsB* gene) and with tRNA^{ileTUV} (encoded by *ileT*) were previously identified and defined by false discovery rate analysis (nucleotide, nt, position are indicated) by Winther et al.[3]

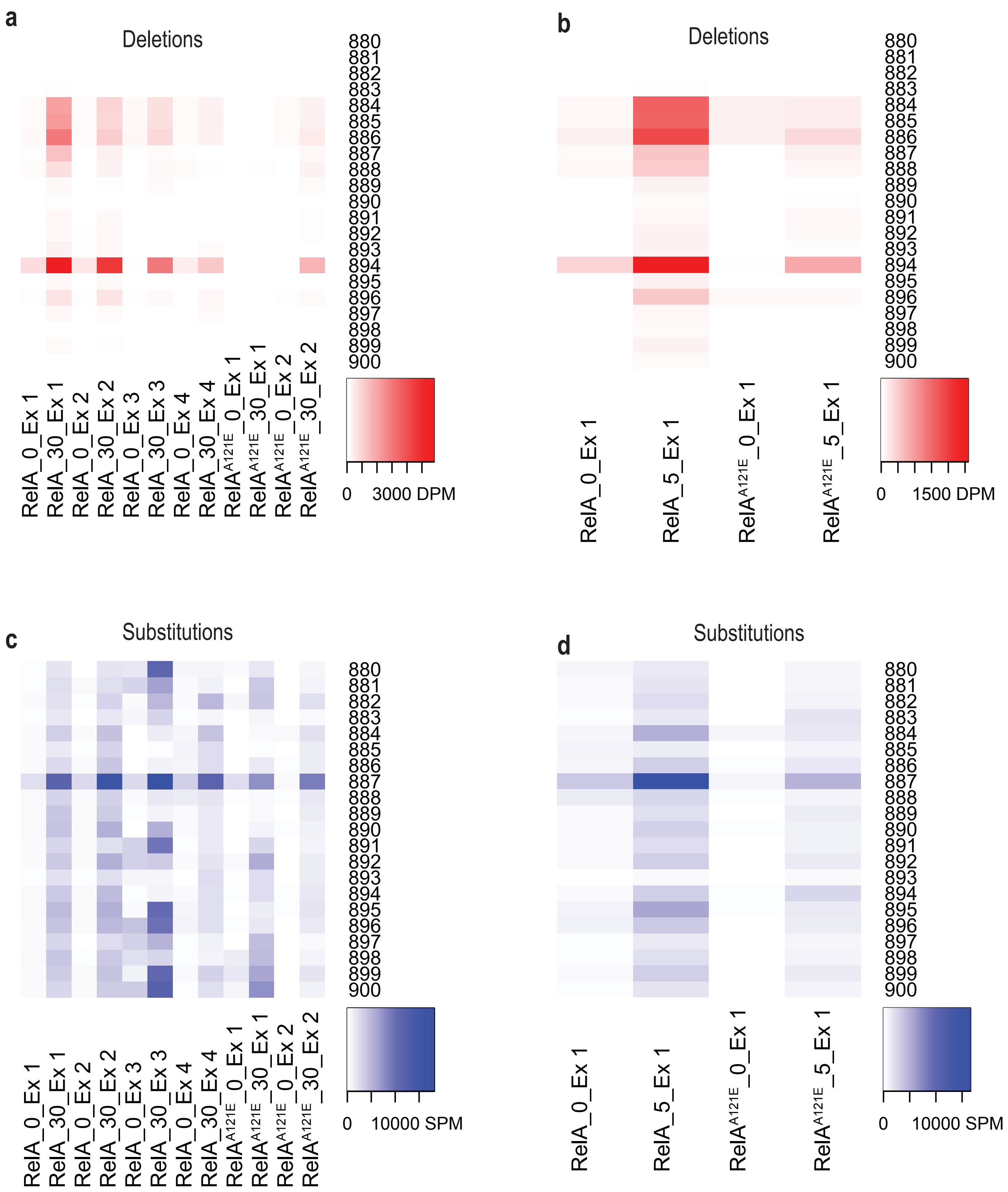

Supplementary Figure 4 - Page 1

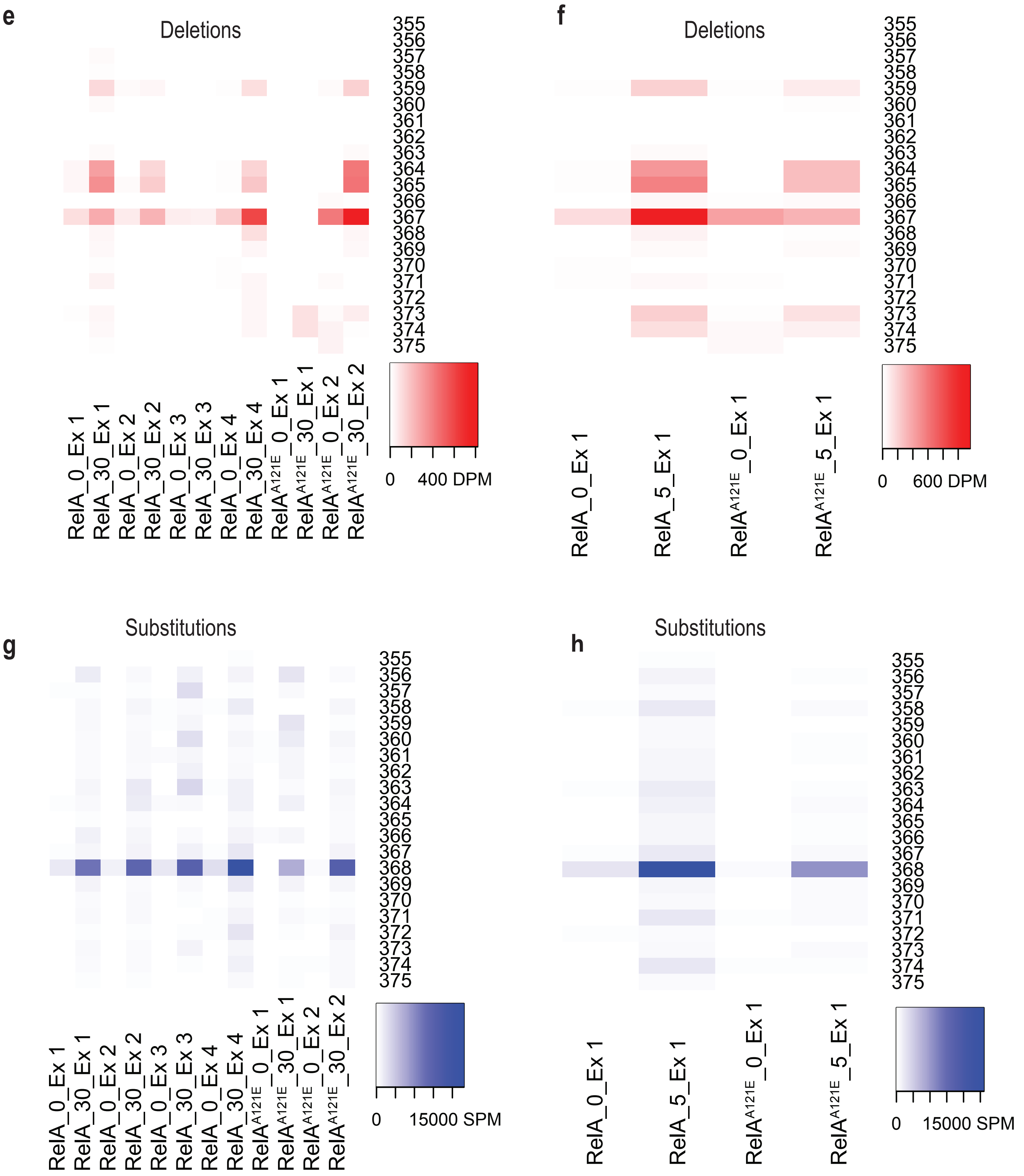
e
Deletions
355 356 357 358 359 360 361 362 363 364 365 366 367 368 369 370 371 372 373 374 375
RelA_0_Ex 1
RelA_30_Ex 1
RelA_0_Ex 2
RelA_30_Ex 2
RelA_0_Ex 3
RelA_30_Ex 3
RelA_0_Ex 4
RelA_30_Ex 4
RelA^{A121E}_0_Ex 1
RelA^{A121E}_30_Ex 1
RelA^{A121E}_0_Ex 2
RelA^{A121E}_30_Ex 2
0 400 DPM
f
Deletions
355 356 357 358 359 360 361 362 363 364 365 366 367 368 369 370 371 372 373 374 375
RelA_0_Ex 1
RelA_5_Ex 1
RelA^{A121E}_0_Ex 1
RelA^{A121E}_5_Ex 1
0 600 DPM
g
Substitutions
355 356 357 358 359 360 361 362 363 364 365 366 367 368 369 370 371 372 373 374 375
RelA_0_Ex 1
RelA_30_Ex 1
RelA_0_Ex 2
RelA_30_Ex 2
RelA_0_Ex 3
RelA_30_Ex 3
RelA_0_Ex 4
RelA_30_Ex 4
RelA^{A121E}_0_Ex 1
RelA^{A121E}_30_Ex 1
RelA^{A121E}_0_Ex 2
RelA^{A121E}_30_Ex 2
0 15000 SPM
h
Substitutions
355 356 357 358 359 360 361 362 363 364 365 366 367 368 369 370 371 372 373 374 375
RelA_0_Ex 1
RelA_5_Ex 1
RelA^{A121E}_0_Ex 1
RelA^{A121E}_5_Ex 1
0 15000 SPM

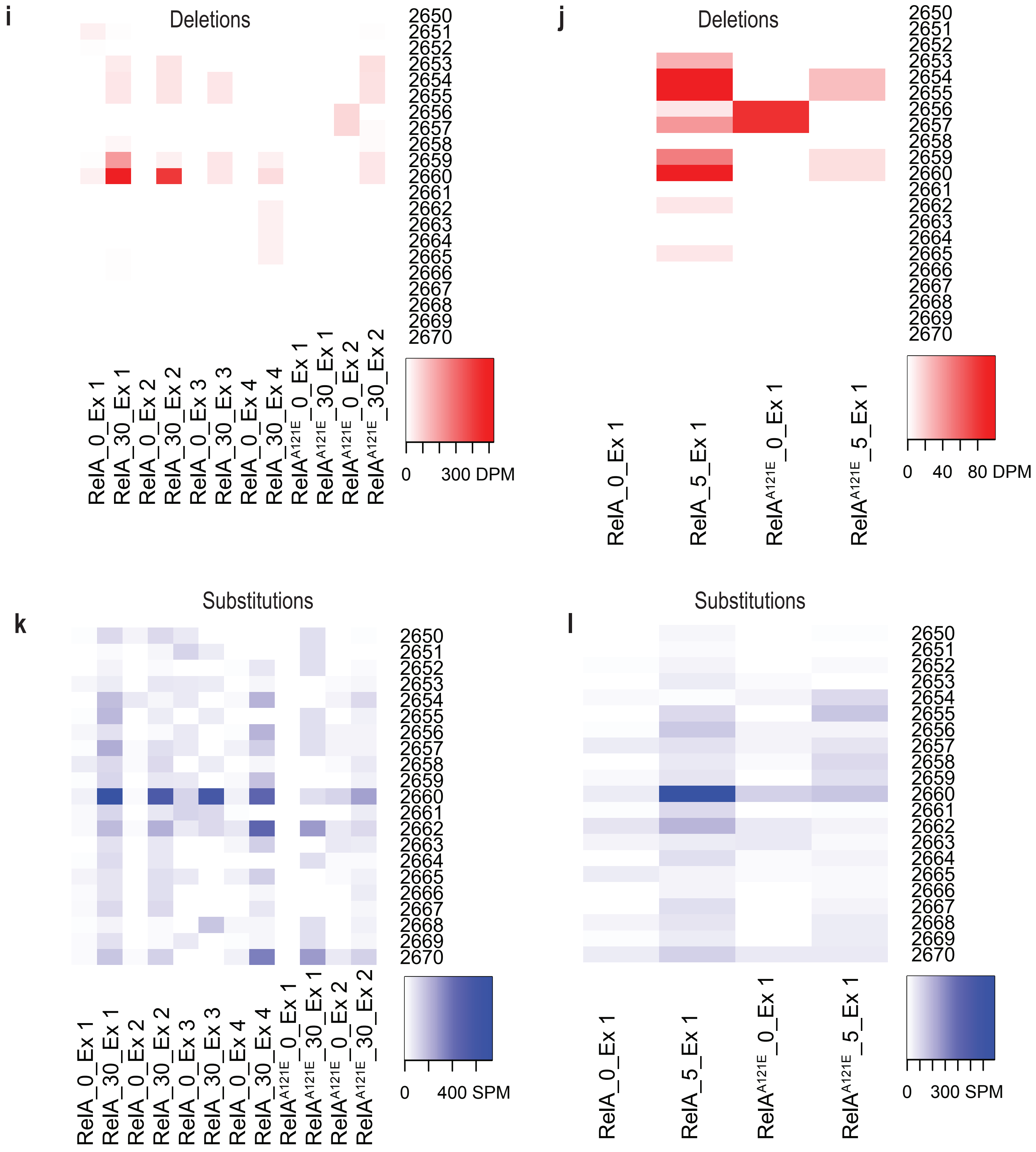
i
Deletions
2650 2651 2652 2653 2654 2655 2656 2657 2658 2659 2660 2661 2662 2663 2664 2665 2666 2667 2668 2669 2670
RelA_0_Ex 1
RelA_30_Ex 1
RelA_0_Ex 2
RelA_30_Ex 2
RelA_0_Ex 3
RelA_30_Ex 3
RelA_0_Ex 4
RelA_30_Ex 4
RelA^{A121E}_0_Ex 1
RelA^{A121E}_30_Ex 1
RelA^{A121E}_0_Ex 2
RelA^{A121E}_30_Ex 2
0 300 DPM
j
Deletions
2650 2651 2652 2653 2654 2655 2656 2657 2658 2659 2660 2661 2662 2663 2664 2665 2666 2667 2668 2669 2670
RelA_0_Ex 1
RelA_5_Ex 1
RelA^{A121E}_0_Ex 1
RelA^{A121E}_5_Ex 1
0 40 80 DPM
k
Substitutions
2650 2651 2652 2653 2654 2655 2656 2657 2658 2659 2660 2661 2662 2663 2664 2665 2666 2667 2668 2669 2670
RelA_0_Ex 1
RelA_30_Ex 1
RelA_0_Ex 2
RelA_30_Ex 2
RelA_0_Ex 3
RelA_30_Ex 3
RelA_0_Ex 4
RelA_30_Ex 4
RelA^{A121E}_0_Ex 1
RelA^{A121E}_30_Ex 1
RelA^{A121E}_0_Ex 2
RelA^{A121E}_30_Ex 2
0 400 SPM
l
Substitutions
2650 2651 2652 2653 2654 2655 2656 2657 2658 2659 2660 2661 2662 2663 2664 2665 2666 2667 2668 2669 2670
RelA_0_Ex 1
RelA_5_Ex 1
RelA^{A121E}_0_Ex 1
RelA^{A121E}_5_Ex 1
0 300 SPM

m

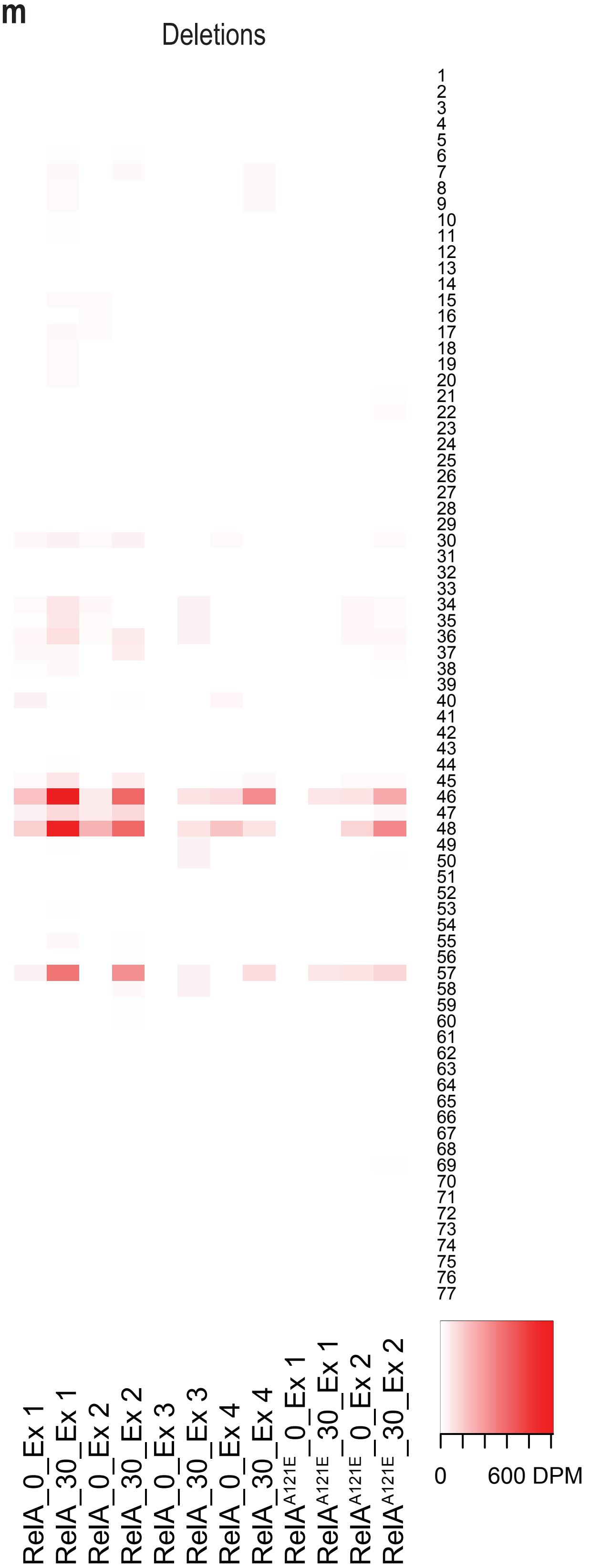
Deletions
1 2 3 4 5 6 7 8 9 10 11 12 13 14 15 16 17 18 19 20 21 22 23 24 25 26 27 28 29 30 31 32 33 34 35 36 37 38 39 40 41 42 43 44 45 46 47 48 49 50 51 52 53 54 55 56 57 58 59 60 61 62 63 64 65 66 67 68 69 70 71 72 73 74 75 76 77
RelA_0_Ex 1
RelA_30_Ex 1
RelA_0_Ex 2
RelA_30_Ex 2
RelA_0_Ex 3
RelA_30_Ex 3
RelA_0_Ex 4
RelA_30_Ex 4
RelA^A121E_0_Ex 1
RelA^A121E_30_Ex 1
RelA^A121E_0_Ex 2
RelA^A121E_30_Ex 2
0
600 DPM

n

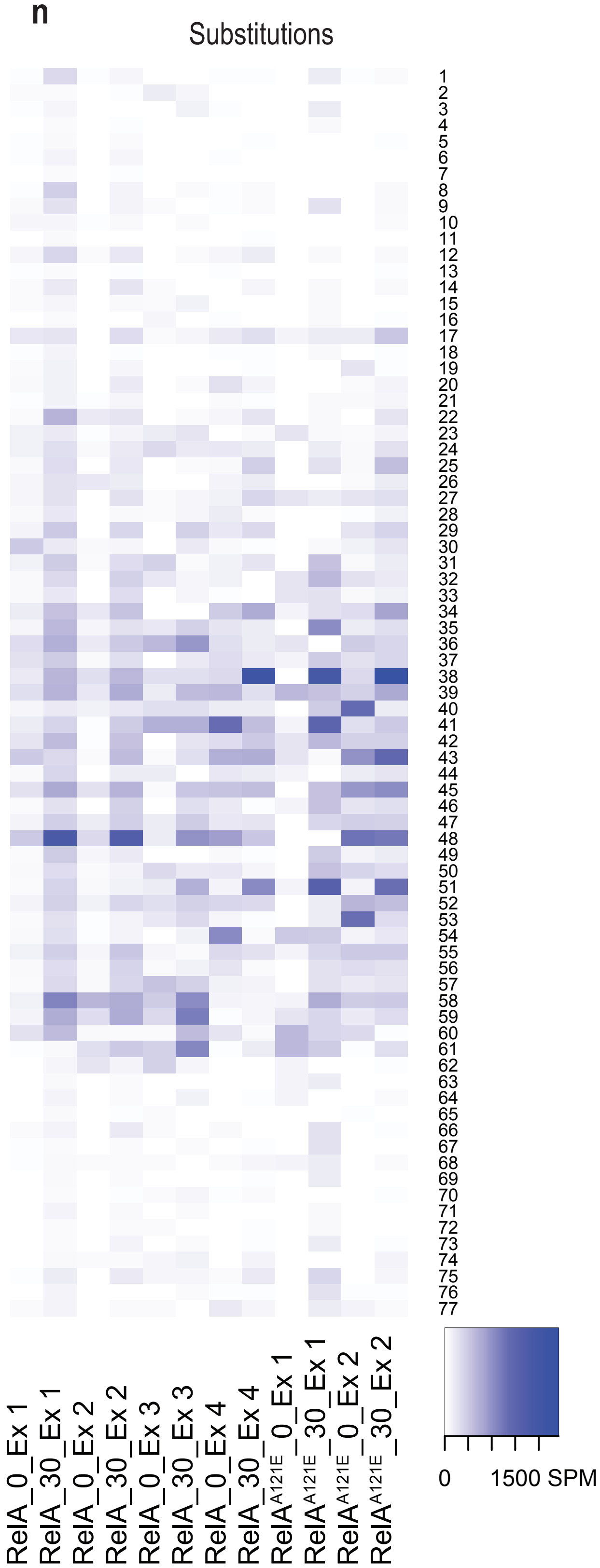
Substitutions
1 2 3 4 5 6 7 8 9 10 11 12 13 14 15 16 17 18 19 20 21 22 23 24 25 26 27 28 29 30 31 32 33 34 35 36 37 38 39 40 41 42 43 44 45 46 47 48 49 50 51 52 53 54 55 56 57 58 59 60 61 62 63 64 65 66 67 68 69 70 71 72 73 74 75 76 77
RelA_0_Ex 1
RelA_30_Ex 1
RelA_0_Ex 2
RelA_30_Ex 2
RelA_0_Ex 3
RelA_30_Ex 3
RelA_0_Ex 4
RelA_30_Ex 4
RelA^A121E_0_Ex 1
RelA^A121E_30_Ex 1
RelA^A121E_0_Ex 2
RelA^A121E_30_Ex 2
0
1500 SPM

Supplementary Figure 4: Interaction-site mapping by cDNA mutational analysis. RT-mutations in cDNA read sequences obtained with CRAC analysis of MG1655 encoding *relA::HTF* or *relA*^{A121E}*::HTF* before (0) and after isoleucine starvation (5 or 30 minutes). **a** and **b** shows Deletions Per Million (DPM in red) cDNA reads mapped in the A-Site Finger (ASF) of 23S rRNA (nt 880-900) after 30 minutes or 5 minutes of starvation. **c** and **d** shows Substitutions Per Million (SPM in blue) mapped to the ASF. **e** and **f** shows DPM in cDNA reads mapped to Helix 15 of 16S rRNA (nt 355-375) after 30 minutes or 5 minutes of starvation. **g** and **h** shows SPM mapped to H15. **i** and **j** shows DPM in cDNA reads mapped to Sarcin-Ricin Loop of 23S rRNA (nt 2650-2670) after 30 minutes or 5 minutes of starvation. **k** and **l** shows SPM mapped to SRL. **m** and **n** shows deletions or substitutions per million in reads aligning to isoleucine tRNA^{ileTUV} (*ileT*) after 30 minutes of starvation.

Supplementary References

1 Blank, K., Hensel, M. & Gerlach, R. G. Rapid and highly efficient method for scarless mutagenesis within the Salmonella enterica chromosome. *PLoS One* **6**, e15763, doi:10.1371/journal.pone.0015763 (2011).

2 Montero, M. *et al.* Systematic production of inactivating and non-inactivating suppressor mutations at the relA locus that compensate the detrimental effects of complete spot loss and affect glycogen content in Escherichia coli. *PLoS One* **9**, e106938, doi:10.1371/journal.pone.0106938 (2014).

3 Winther, K. S., Roghanian, M. & Gerdes, K. Activation of the Stringent Response by Loading of RelA-tRNA Complexes at the Ribosomal A-Site. *Mol Cell* **70**, 95-105 e104, doi:10.1016/j.molcel.2018.02.033 (2018).

4 Sinha, A. K., Winther, K. S., Roghanian, M. & Gerdes, K. Fatty acid starvation activates RelA by depleting lysine precursor pyruvate. *Mol Microbiol* **112**, 1339-1349, doi:10.1111/mmi.14366 (2019).
