# Supplementary material for: The RelA hydrolase domain acts as a molecular switch for (p)ppGpp synthesis
Source: Commun Biol. 2021 Mar 31;4:434. doi: 10.1038/s42003-021-01963-z (PMC8012599; doi:10.1038/s42003-021-01963-z)
Supplement: Supplementary file 3 — Reporting Summary [file 42003_2021_1963_MOESM3_ESM.pdf]

nature research

Corresponding author(s): KristofferSkovbo Winther

Last updated by author(s): Feb 12, 2021

Reporting Summary

Nature Research wishes to improve the reproducibility of the work that we publish. This form provides structure for consistency and transparency in reporting. For further information on Nature Research policies, see our Editorial Policies and the Editorial Policy Checklist.

Statistics

For all statistical analyses, confirm that the following items are present in the figure legend, table legend, main text, or Methods section.

n/a	Confirmed	
☐	☒	The exact sample size (*n*) for each experimental group/condition, given as a discrete number and unit of measurement
☐	☒	A statement on whether measurements were taken from distinct samples or whether the same sample was measured repeatedly
☒	☐	The statistical test(s) used AND whether they are one- or two-sided<br>*Only common tests should be described solely by name; describe more complex techniques in the Methods section.*
☒	☐	A description of all covariates tested
☒	☐	A description of any assumptions or corrections, such as tests of normality and adjustment for multiple comparisons
☐	☒	A full description of the statistical parameters including central tendency (e.g. means) or other basic estimates (e.g. regression coefficient) AND variation (e.g. standard deviation) or associated estimates of uncertainty (e.g. confidence intervals)
☒	☐	For null hypothesis testing, the test statistic (e.g. *F*, *t*, *r*) with confidence intervals, effect sizes, degrees of freedom and *P* value noted<br>*Give P values as exact values whenever suitable.*
☒	☐	For Bayesian analysis, information on the choice of priors and Markov chain Monte Carlo settings
☒	☐	For hierarchical and complex designs, identification of the appropriate level for tests and full reporting of outcomes
☒	☐	Estimates of effect sizes (e.g. Cohen's *d*, Pearson's *r*), indicating how they were calculated

Our web collection on statistics for biologists contains articles on many of the points above.

Software and code

Policy information about availability of computer code

Data collection	No software was used for data collection
Data analysis	Barcode filtering/demultiplexing software:<br>pyBarcodeFilter.py (v2.3.4) Webb et al., 2014 https://bitbucket.org/sgrann/pycrac<br>Read trimming software:<br>Cutadapt (v1.4.2) http://journal.embnet.org/index.php/embnetjournal/article/view/200 https://cutadapt.readthedocs.io/en/stable/<br><br>Software for removal of PCR duplicates:<br>pyFastqDuplicateRemover.py (v0.0.2) Webb et al., 2014 https://bitbucket.org/sgrann/pycrac<br><br>Read alignment software:<br>Bowtie2 (v2.1.0) Langmead and Salzberg, 2012 http://bowtie-bio.sourceforge.net/bowtie2/index.shtml<br><br>Data and mutational analysis software:<br>pyReadCounter.py (v0.5.3) Webb et al., 2014 https://bitbucket.org/sgrann/pycrac<br><br>Data processing is also described in the GEO database repository and has also been published previously in (Winther et al. 2018)

For manuscripts utilizing custom algorithms or software that are central to the research but not yet described in published literature, software must be made available to editors and reviewers. We strongly encourage code deposition in a community repository (e.g. GitHub). See the Nature Research guidelines for submitting code & software for further information.

Data

Policy information about availability of data

All manuscripts must include a data availability statement. This statement should provide the following information, where applicable:

- Accession codes, unique identifiers, or web links for publicly available datasets
- A list of figures that have associated raw data
- A description of any restrictions on data availability

Sequencing data obtained here has been deposited with GEO accession GSE150416
Sequencing data analysed in samples RelA_0_Ex1, RelA_30_Ex1, RelA_0_Ex2 and RelA_30_Ex2 can be accessed with GEO accession: GSM2912989, GSM2912991, GSM2912990 and GSM2912992.

Fig. 3b, 4a-f, Supplementary Figure 3b-h and 4a-n are derived from associated raw data

There is no restriction on data availability.

Field-specific reporting

Please select the one below that is the best fit for your research. If you are not sure, read the appropriate sections before making your selection.

☒ Life sciences ☐ Behavioural & social sciences ☐ Ecological, evolutionary & environmental sciences

For a reference copy of the document with all sections, see nature.com/documents/nr-reporting-summary-flat.pdf

Life sciences study design

All studies must disclose on these points even when the disclosure is negative.

Sample size	At least two independent biological replicates
Data exclusions	No data was excluded
Replication	Reproducibility of the experiments were verified by the growth experiments, ppGpp measurements and by CRAC analysis. We confirm that results were highly reproducible.
Randomization	This is not relevant for our study because we have not done sampling study here. We have done genetic experiments on E. coli. We generated mutants, characterized their phenotypes and analysed properties of mutant proteins in vivo.
Blinding	Blinding was not relevant to our study because we were collecting data from wild type and mutant E. coli strains. All data were compared between wild type and mutants.

Reporting for specific materials, systems and methods

We require information from authors about some types of materials, experimental systems and methods used in many studies. Here, indicate whether each material, system or method listed is relevant to your study. If you are not sure if a list item applies to your research, read the appropriate section before selecting a response.

Materials & experimental systems

n/a	Involved in the study
☒	☐ Antibodies
☒	☐ Eukaryotic cell lines
☒	☐ Palaeontology and archaeology
☒	☐ Animals and other organisms
☒	☐ Human research participants
☒	☐ Clinical data
☒	☐ Dual use research of concern

Methods

n/a	Involved in the study
☒	☐ ChIP-seq
☒	☐ Flow cytometry
☒	☐ MRI-based neuroimaging
