# Supplementary material for: The RelA hydrolase domain acts as a molecular switch for (p)ppGpp synthesis
Source: Commun Biol. 2021 Mar 31;4:434. doi: 10.1038/s42003-021-01963-z (PMC8012599; doi:10.1038/s42003-021-01963-z)
Supplement: Supplementary file 1 — Peer Review File [file 42003_2021_1963_MOESM1_ESM.pdf]

Reviewers' comments:

Reviewer #1 (Remarks to the Author):

In this report Sinha and Winter study the role of a loop (dubbed the 'H loop') element in enzymatic competence of Escherichia coli RelA-SpoT Homolog enzyme RelA. The loop is part of the catalytically inactive pseudo-hydrolase domain, pseudo-HD, of the RelA factor, and since mutations in the H-loop compromise the synthesis activity of the enzyme (which is mediated by the catalytically-competent (p)ppGpp-synthetase domain, SYNTH), the authors conclude that the loop is a key regulator of the SYNTH. Importantly, the fact that mutations in pseudo-HD can compromise the SYNTH activity was reported earlier and is not novel (Montero et al. PLOS ONE 2014, ref. 33).
Unfortunately, the results presented in the paper are not sufficient to arrive at this conclusion. I do not feel the level of rigour is sufficient to motivate the publication in Communications Biology. However, since the data are otherwise solid, I would recommend stripping the text of over-interpretations and publishing the data in more general NPG journal, Scientific Reports.

Specific comments:

1. Lines 39-40: 'GDP/GTP analogs, guanosine tetra- and pentaphosphate (collectively referred to as (p)ppGpp or alarmones)'. ppGpp and pppGpp are not, chemically speaking, analogues of GDP and GTP; e.g. ppApp / pppApp are. Please amend.
2. Lines 46-47: 'The RelA protein has only (p)ppGpp synthetase activity but carries an inactive pseudo-hydrolase domain, whereas, SpoT is a weak (p)ppGpp synthetase and exhibits strong hydrolase activity.' The reference is missing.
3. 'These complexes are thought to be loaded at empty ribosomal A-sites during amino acid starvation, when EF-Tu•GTP•tRNA ternary complexes are scarce. Such complexes have also been reported in vitro using a different methodology22.' First, this section is irrelevant for the current study. Second, it portrays the results published by the authors earlier as the final, undisputed truth. This is not the case. The complex formation between RelA and tRNA was not detected in 22 unless the crosslinker was used. Please specify that. Similarly, no complex was detected by Kurdin and colleagues (PMID: 29390134). A follow-up from Takada and colleagues strongly argued against the tRNA delivery model (https://www.biorxiv.org/content/10.1101/2020.01.17.910273v1). Moreover, Loveland and colleagues solved by cryo-EM the structure of RelA bound to vacant A-site (no tRNA) and argued that RelA binding to the ribosome precedes tRNA recruitment (PMID: 27434674). Please either discuss the topic adequately or remove the section – I recommend the latter given that it is completely irrelevant for the current paper.
4. Lines 71-73: 'The switch-ON signal for bifunctional enzymes is still not clear, but for Rel proteins, (p)ppGpp synthesis results from an accumulation of uncharged tRNA during amino acid starvation26-28' Please rework. I guess the 'bifunctional enzymes' in question are SpoT, since bifunctional Rel are activated by starved ribosomes, right?
5. Line 79: 'E. coli RelA contains a HYD domain': pseudo-HD, please specify that.
6. Lines 109-110: 'Interestingly, the alignment revealed a short region (Residues 114-130) in the HYD domain between α6 and α7 unique only to RelA homologs (Fig. 1B and Fig. S1D).' The full-size alignment of the pseudo-HD makes it clear that there are other loop segments with similar patterns of lack of conservation (SFig 1). The authors are following up on the reported mutation novel (Montero et al. PLOS ONE 2014) and motivate their focus by conservation studies. This is not really working. If the H-loop is special, the authors should demonstrate that experimentally by applying similar mutagenesis screens to other unconserved loop regions. I bet that substitutions that disrupt these regions (such as for Pro) will have similar effects. Please either do not stipulate that the H-loop in any way special and just spell it out that this is what was done because of the earlier report Montero et al. PLOS ONE 2014 or perform the additional random mutagenesis study to demonstrate that the H-loop is more crucial

that other unconserved loops. For starters, one could just chop off the extra N-terminal 5-10 amino acids. Let us see if this would kill RelA (I have a hunch it will; adding N-terminal 6His already messes up the protein quite a lot… no idea why!).
7. Note that the H-loop is shorter and is different in sequence in Rel. Rel is activated by starved ribosomes, just like RelA, and HD and SYNTH regulate each other – i.e. RelA and RelA are likely to share the key regulatory mechanisms. If the H-loop would be 'regulatory', it would be conserved and present in both. While it is clear that RelA can me inactivated by mutations in the pseudo-HD, this does not suggest that we are dealing with a conserved dedicated mechanism: it is easy to break the protein, especially when it is such as delicate one as RelA, which is regulated by a multi-layered mechanism.
8. Line 115: 'For simplicity we refer to this loop as the H-loop (Hydrolase-loop).' The are many loops in the pseudo-HD, and it is unclear as to why the H-loop is special. Please specify the position, 'alpha6-alpha7 H-loop'. This puts the loop on the structure and one can then compare the results for different H-loops. Right now giving the loop a name singles it out of the other H-loops and makes it more functionally important; no functional data suggest that it is.
9. Line 119: 'Indeed, deletion of a part of the H-loop (Δ116-129)'. In the absence of dedicated assays demonstrating that the deletion does not affect the protein structure it is hard to say anything about this loss-of-function mutant: I would guess that deletion of 14 amino acids at random in HD domain is likely to kill RelA's SYNTH activity. In general, the authors never assess the protein folding / structure upon introduction of the mutations / deletions, e.g. by CD spectrum analysis of purified mutants. In the absence of biochemical results, it is impossible to assess the mechanistic effects of the mutants.
10. Line 130: 'We conclude that the H-loop of inactive HYD domain of RelA regulates (p)ppGpp synthesis.' This conclusion is not based on the results presented. What one conclude is that mutating the loop one can inhibit the SYNTH activity. Regulation is implied by the authors but there is no reason to believe that.
11. Lines 308-309: 'Another possibility is that the (p)ppGpp synthesis could stabilize the binding of RelA to the A-site and promote increased synthesis by a mechanism similar to positive allosteric feed-back36,38.' Recently Takada and collegues specifically investigate the effects of ppGpp / synthesis activity on the interaction of B. subtilis Rel. Please incorporate this paper into your discussion.
12. Lines 312-303: 'In conclusion, we present robust evidence demonstrating that the inactive HYD domain of RelA plays a novel regulatory role in controlling (p)ppGpp synthetase activity of the SYN domain.' I am not sure that this is a novel conclusion. Clearly, the pseudo-HD is crucial for activity of RelA, given that it is conserved and retained though evolution. Mutations in this domain were shown earlier to abrogate the enzymatic activity (i.e. toxicity in ppGpp0 background, Montero et al. PLOS ONE 2014). Here the authors confirm these results but do not provide a mechanistic explanation.
13. Figure 1c: please use the structure from Tamman et al. (Rel complexed with native ppGpp) instead of the currently used (with pGpp).

Reviewer #2 (Remarks to the Author):

This manuscript describes is an important discovery regarding some very old questions. One is why the RelA protein E. coli and other Graham negative bacteria persist in evolution with a ppGpp synthetase but an inactive hydrolase but retains much of the sequence of an active domain. Most other bacteria have instead a bifunctional RSH gene with sequence homology with RelA. Both are able to self-regulate and balance an otherwise opposing activities to avoid an energy drain from a futile cycle of synthesizing (p)ppGpp and degrading it to GTP and GTP. How does RelA, a monofunctional (p)ppGpp synthetase regulates its synthesis activity without hydrolysis activity like most RSH proteins? The authors here describe the answer with a series of convincing experiments using RelA

hypomorph mutants that define a 16-residue loop in the hydrolase-inactive domain that diminish ribosomal binding and manner independent of changing tRNA binding to ribosomes. Localization of effects of loop contacts on ribosomes is documented by an elegant sequencing methodology worked out earlier for mapping protein-RNA contacts by increased point mutants and deletions in rRNA. Alignments of this region from many strains of this loop confirms its absence in RSH enzymes and presence in several Grahm negative RelA strains.

Critique
This work is clearly presented in a logical manner with convincing data. Publication is recommended. I have only minor problems with the manuscript.

It is still puzzling to me that Δ116-129 (H-loop) increases SMG sensitivity & lowers (p)ppGpp accumulation (Fig 1) as if needed for synthesis activity whereas the quad mutants that neutralize charge and are plausibly argued to alter H-loop structure increases (p)ppGpp and SMG resistance above WT. On the surface this dilemma could be taken to argue the H-loop only provides a spacer function; interestingly the point mutants that lower ppGpp levels and SMG sensitivity argue against a simple spacer function.
There are two future experiments that might be interesting…just musing and not needed in this work to be published! One is to substitute the RelA H-loop for the missing H-loop in a well studied RelSeq RSH gene hydrolase/synthetase fragment lacking the C-terminal domain and see what happens. Also it might be interesting to turn the crank again and screen for mutants in a relA H-loop deletion with little synthetic activity and again screen for SMG-r mutants. Also screen for RelA quad mutants for slow growing suppressors.
Finally, Figure 4 makes it seem like the A121 residue is a very long way from the most mutagenic 2660 residue in the SRL domain.

Figures: 1. All have panel labels are are placed so that they disappear when printed.
2. Figs 1 & 2 label SMG resistance as "functional". SMG resistance is more accurate.
3. 3-aminotriazole resistance on plates containing all AA-his provides another (less sensitive) measure of increased (p)ppGpp levels. The expectation is that weak ppGpp elevation would not be sensitive and high elevation would be resistant.

Line 132 inhibits should be singular… mutations inhibit.

Reviewer #3 (Remarks to the Author):

In the article by Sinha and Winther submitted to Communications Biology the authors investigate the regulatory role of a short loop in the tertiary structure of RelA, a conserved synthetase of guanosine penta and tetraphosphate (p)ppGpp, two stress response alarmones that cue physiological adaptation of bacteria. RelA is important for antimicrobial resistance and bacterial virulence gene regulation and pathogenesis. In general, bacteria have one enzyme, Rel/Spo, which acts as a both a synthetase and hydrolase of (p)ppGpp. Typically, the hydrolase domain (HYD) of the bifunctional enzyme is essential for bacterial growth and viability in the context of an intact synthetase and it has been shown that the hydrolase activity in the bifunctional enzyme is an important regulator of synthetase activity. For E. coli and most gamma proteobacteria, the hydrolase domain of RelA is degenerate and non-functional as a hydrolase of (p)ppGpp. These bacteria encode a second bifunctional enzyme known as SpoT. The authors test the hypothesis that specific residues and secondary structures within the HYD of RelA are necessary for regulating synthetase activity of the enzyme in organisms that encode RelA and SpoT. They provide evidence in support of their prediction and use sequence alignments and a published

crystal structure of the RelA-tRNA-ribosome complex to identify a short ~15 residue loop in the HYD, which is absent in hydrolase active Rel/Spo proteins. The authors employ cutting edge site-directed chromosomal mutagenesis of E. coli to generate a loop deletion mutant, and screen for substitutions within the loop that perturb RelA synthetase activity. They use assays of bacterial viability during amino acid starvation and biochemical measurements of (p)ppGpp to show that the loop and particular residues within the loop are necessary for promoting the synthetase activity of RelA. They expand their observations by using chemical crosslinking and mass spectrometry to probe the effects of the loop mutants on complex formation. They define differences in the degree of interaction between RelA and the ribosome that might explain the change in synthetase activity conveyed by perturbing the regulatory loop in the HYD.

This is a highly rigorous examination of the role of the HYD loop in the regulation of RelA, a critical protein for bacteria. The work is comprehensive and focused. It is fully appropriate for the mission and scope of this journal and should be broadly read. The use of site directed substitution mutagenesis and genetic screening to define structure function principals is exemplary. Also, the chemical crosslinking and mass spectrometry to define differences in interactions within the RelA-ribosome complex was impressive, especially since they included 5 min and 30 min post starvation time points and measured the interactions in bacteria cells expressing wild type and mutant proteins. I recommend that the work be accepted with minor revisions. I offer only minor critiques and criticisms that might make the work more coherent and comprehendible for naïve audiences.

Semi Major Critique.

Other than western blots, which were hard to interpret, there is no measurement of protein folding for the tagged RelA variants. Something like, circular dichroism or tryptophan fluorescence could be conducted. Perhaps the cross-linking somehow shows this already. If so, than it should be stated explicitly.

Minor Critiques

Line 39 Are ppGpp and pppGpp truly "analogs" of GDP/GTP? Perhaps a different adjective is needed.

Line 54 "hungry" is not an appropriate term for a ribosome. Did you mean "apo" in line 65 empty is used, this might be better?

Line 103 SMG plates is not appropriately introduced or discussed as a RelA activity assay. Please explain how this biological assay works early in the results.

Lines 106-108 Not sure why the specific results of this study are stated, I suggest removing these sentences and replace with a description of SMG plates

Line 114 What is Relseq?

The acronyms become really brutal for a naïve reader, is HTF necessary in the main text. Is this the tag? Can it be removed or simplified?

Line 538 The title of this figure does not read well. Perhaps something like "Residues of 114-130 of the RelA pseudo-hydrolase domain form a loop that controls ppGpp synthesis"

Line 558 To clarify, can you briefly explain why L-Valine prompts isoleucine starvation?

Line 570-571 Please cite the Salmonella PLOS-One paper that was the first to use this method in the main text and in this legend?

Response to referees' comments, 25th November 2020

Reviewer #1 (Remarks to the Author):

In this report Sinha and Winter study the role of a loop (dubbed the 'H loop') element in enzymatic competence of Escherichia coli RelA-SpoT Homolog enzyme RelA. The loop is part of the catalytically inactive pseudo-hydrolase domain, pseudo-HD, of the RelA factor, and since mutations in the H-loop compromise the synthesis activity of the enzyme (which is mediated by the catalytically-competent (p)ppGpp-synthetase domain, SYNTH), the authors conclude that the loop is a key regulator of the SYNTH. Importantly, the fact that mutations in pseudo-HD can compromise the SYNTH activity was reported earlier and is not novel (Montero et al. PLOS ONE 2014, ref. 33).
Unfortunately, the results presented in the paper are not sufficient to arrive at this conclusion. I do not feel the level of rigour is sufficient to motivate the publication in Communications Biology. However, since the data are otherwise solid, I would recommend stripping the text of over-interpretations and publishing the data in more general NPG journal, Scientific Reports.

We thank reviewer for the summary and pointing out the solidity of our work. We agree that Montero et al. PLOS ONE 2014 have reported some of the mutations in RelA that compromise the synthetase activity and therefore we have cited this study in our manuscript.

We politely disagree with the reviewer that our finding is not novel. The real novelty of our data is the identification of the unique extended H-loop, which, according to our results, plays a crucial role in regulating synthetase activity and is only present in RelA homologs. This is the first report where we show that a specific site in RelA pseudo-HD can compromise the synthetase activity. Interestingly, some of the mutations, reported by Montero et al. PLOS ONE 2014, such as I116L and Q131L are in or flanking this H-loop further strengthens our hypothesis that the H-loop is important for regulation of SYN domain and synthetase activity. The other mutations reported by Montero et al. PLOS ONE 2014 including ΔW39, which, according to us, can drastically affect protein's stability and structure thus will not be very conclusive. Furthermore, we have identified a single mutation A121E in the H-loop that is important for regulating synthetase activity without compromising $RelA^{A121E}$ protein's binding conformation to ribosome. In addition, we have identified stimulating mutations in the same loop that increases (p)ppGpp synthesis.

Therefore, our study forwards our understanding and indicates an important mechanistic detail of hydrolase domain regulating synthetase domain of RelA.

Specific comments:

1) Lines 39-40: 'GDP/GTP analogs, guanosine tetra- and pentaphosphate (collectively referred to

as (p)ppGpp or alarmones)'. ppGpp and pppGpp are not, chemically speaking, analogues of GDP and GTP; e.g. ppApp / pppApp are. Please amend.

We agree. "Specialized GTP/GDP analogs" has been removed. The sentence now reads:" Derivatives of GDP/GTP, guanosine tetra- and pentaphosphate (collectively referred to as (p)ppGpp or alarmones), are the effector molecules of the stringent response and are synthesized/hydrolyzed by the RSH superfamily (RelA/SpoT homologues) proteins."(line 39)

2) Lines 46-47: 'The RelA protein has only (p)ppGpp synthetase activity but carries an inactive pseudo-hydrolase domain, whereas, SpoT is a weak (p)ppGpp synthetase and exhibits strong hydrolase activity.' The reference is missing.

We have included the missing reference (line 47).

3) 'These complexes are thought to be loaded at empty ribosomal A-sites during amino acid starvation, when EF-Tu•GTP•tRNA ternary complexes are scarce. Such complexes have also been reported in vitro using a different methodology22.' First, this section is irrelevant for the current study. Second, it portrays the results published by the authors earlier as the final, undisputed truth. This is not the case. The complex formation between RelA and tRNA was not detected in 22 unless the crosslinker was used. Please specify that. Similarly, no complex was detected by Kurdin and colleagues (PMID: 29390134). A follow-up from Takada and colleagues strongly argued against the tRNA delivery model (https://www.biorxiv.org/content/10.1101/2020.01.17.910273v1). Moreover, Loveland and colleagues solved by cryo-EM the structure of RelA bound to vacant A-site (no tRNA) and argued that RelA binding to the ribosome precedes tRNA recruitment (PMID: 27434674). Please either discuss the topic
adequately or remove the section – I recommend the latter given that it is completely irrelevant for the current paper.

We agree that it is not important for the current study therefore, the section has now been rephrased.

The section now reads (Lines 64-65): "RelA is thought to bind with tRNA at ribosomal A-sites during amino acid starvation, when EF-Tu•GTP•tRNA ternary complexes are scarce".

We would, however, like to point out that while relA-tRNA interaction was not observed without crosslinker by EMSAs in reference 22, which was also the case in the studies of Kudrin and Takada et al. RelA-tRNA interaction was observed with a more sensitive flourometric method in reference 22. Furthermore, ribosome independent Rel-tRNA interaction has also more recently been reported in Pausch et al. 2020. (https://doi.org/10.1016/j.celrep.2020.108157).

4) Lines 71-73: 'The switch-ON signal for bifunctional enzymes is still not clear, but for Rel proteins, (p)ppGpp synthesis results from an accumulation of uncharged tRNA during amino acid starvation26-28' Please rework. I guess the 'bifunctional enzymes' in question are SpoT, since bifunctional Rel are activated by starved ribosomes, right?

We agree. "bifunctional enzymes" has been replaced with "SpoT" (line 73)

5) Line 79: 'E. coli RelA contains a HYD domain': pseudo-HD, please specify that. We agree. To clarify this we have changed "HYD domain" to "pseudo-HD" in the manuscript.

6) Lines 109-110: ‘Interestingly, the alignment revealed a short region (Residues 114-130) in the HYD domain between α6 and α7 unique only to RelA homologs (Fig. 1B and Fig. S1D).’ The full-size alignment of the pseudo-HD makes it clear that there are other loop segments with similar patterns of lack of conservation (SFig 1). The authors are following up on the reported mutation novel (Montero et al. PLOS ONE 2014) and motivate their focus by conservation studies. This is not really working. If the H-loop is special, the authors should demonstrate that experimentally by applying similar mutagenesis screens to other unconserved loop regions. I bet that substitutions that disrupt these regions (such as for Pro) will have similar effects. Please either do not stipulate that the H-loop in any way special and just spell it out that this is what was done because of the earlier report Montero et al. PLOS ONE 2014 or perform the additional random mutagenesis study to demonstrate
that the H-loop is more crucial that other unconserved loops. For starters, one could just chop off the extra N-terminal 5-10 amino acids. Let us see if this would kill RelA (I have a hunch it will; adding N-terminal 6His already messes up the protein quite a lot... no idea why!).

The study by Montero et al. used random mutagenesis in the full-length RelA protein, which allows deletion of otherwise essential SpoT protein. The majority of mutations were expectedly found in the synthetase domain, whereas four mutations were located in the pseudo-hydrolase domain. Of these four pseudo-hydrolase mutations: one is a deletion (ΔW39), which is also used in our study, is likely to result major structural change in the N-terminal domain and therefore, was not investigated further. Consistently, substitution of the residue W39A (tryptophan to an alanine substitution) did not affect RelA activity in our study (Figure S1C).

The second reported mutation in Montero et al. is R96P. Proline substitutions can induce major effects on protein structure. We mutated R96A (Figure S1C) but did not see any effect, this suggests it is the proline substitutions that might be deleterious for the domain structure. The final two mutations in the N-terminal pseudo-hydrolase domain reported by Montero et al. is I116L and Q131L. Both mutations encompass the H-loop and therefore prompted us to identify the importance of this loop. I116L is an extremely subtle change and we also confirmed here that this subtle substitution indeed affects RelA activation (Figure S2). The loop is observed to be located in a part of the hydrolase domain, which contains the ppGpp binding site and moves during switching between hydrolase/synthase activities, this is consistent with its role in RelA activation. In addition the loop is observed to be longer in hydrolase inactive RelA (Figure 1A and B) as compared to SpoT/Rel and finally we can also introduce mutations in this loop that increase RelA activation (Figure 5). In conclusion this loop does appear to be special.

With the information presented here, it is expected that N-terminal tagging of RelA or removal 5-10 amino acid could affect the overall structure of the HD and therefore also the switching mechanism and activation of RelA.

7) Note that the H-loop is shorter and is different in sequence in Rel. Rel is activated by starved ribosomes, just like RelA, and HD and SYNTH regulate each other – i.e. RelA and RelA are likely to share the key regulatory mechanisms. If the H-loop would be ‘regulatory’, it would be conserved and present in both. While it is clear that RelA can me inactivated by mutations in the pseudo-HD, this does not suggest that we are dealing with a conserved dedicated mechanism: it is easy to break the protein, especially when it is such as delicate one as RelA, which is regulated by a multi-layered mechanism.

While RelA and Rel both are activated by amino acid starvation and the overall structure of the complex with the ribosome is very similar (recently observed in Pausch et al 2020), RelA does not hydrolyze (p)ppGpp and does not contain the conserved residues for (p)ppGpp binding and catalysis. On the other hand RelA pseudo-HD is larger than the active hydrolase domain of Rel proteins (Figure S1D), which suggests that perhaps in RelA the hydrolase has evolved for its regulatory function. Strikingly the H-loop is extended in RelA proteins and located at the site of (p)ppGpp binding in active hydrolase domain in Rel protiens (Figure 1C). In RelSeq, which was included in the previous version of the manuscript the loop is predicted to directly overlap with the (p)ppGpp binding site. As we know that (p)ppGpp binding to the hydrolase domain results in large structural changes it is also very likely that movement of the H-loop will directly regulate the synthase domain.

We did perform random mutagenesis screen to avoid "breaking" the protein. Furthermore, we did alanine scan of other sites in the hydrolase domain, which did not affect the activity of the protein (Figure S1).

8) Line 115: 'For simplicity we refer to this loop as the H-loop (Hydrolase-loop).' The are many loops in the pseudo-HD, and it is unclear as to why the H-loop is special. Please specify the position, 'alpha6-alpha7 H-loop'. This puts the loop on the structure and one can then compare the results for different H-loops. Right now giving the loop a name singles it out of the other H-loops and makes it more functionally important; no functional data suggest that it is.

We thank the reviewer for this suggestion. We have mentioned it as (between α6 and α7) in line-90. To make this even clearer it has now also been indicated in Figure 1B. We agree that there are other smaller loops in the RelA pseudo-HD however; none of them is as conserved and distinct as this loop. Our results provide a solid evidence that this loop is functionally important for RelA SYNTH activity. Therefore, we would like to keep the name as H-loop for simplicity here.

9) Line 119: 'Indeed, deletion of a part of the H-loop (Δ116-129)'. In the absence of dedicated assays demonstrating that the deletion does not affect the protein structure it is hard to say anything about this loss-of-function mutant: I would guess that deletion of 14 amino acids at random in HD domain is likely to kill RelA's SYNTH activity. In general, the authors never assess the protein folding / structure upon introduction of the mutations / deletions, e.g. by CD spectrum analysis of purified mutants. In the absence of biochemical results, it is impossible to assess the mechanistic effects of the mutants.

(Δ116-129) was only assessed for primary screening purposes. We agree with the reviewer and we therefore conducted a screen to identify single point-mutations that are less likely to affect overall protein folding (Figure 2).

Nevertheless, we would like to point out that our CRAC experiments clearly reveal that there is no perturbation in the structure when we have mutated a residue $RelA^{A121E}$ in the H-loop. The binding pattern to tRNA and to ribosome of this mutant is exactly similar to the wild type protein. Thus, we agree that we don't have data to show how the deletion (Δ116-129) affects protein stability however, the deletion is our primary screening experiment but our major conclusion for the importance of this region is based on the point mutations located in this region, especially $RelA^{A121E}$.

10) Line 130: ‘We conclude that the H-loop of inactive HYD domain of RelA regulates (p)ppGpp synthesis.’ This conclusion is not based on the results presented. What one conclude is that mutating the loop one can inhibit the SYNTH activity. Regulation is implied by the authors but there is no reason to believe that.

We agree. The sentence has been rephrased and now reads:” In conclusion these results reveal a role of the H-loop in RelA activation (line 136-137).

11) Lines 308-309: ‘Another possibility is that the (p)ppGpp synthesis could stabilize the binding of RelA to the A-site and promote increased synthesis by a mechanism similar to positive allosteric feed-back36,38.’ Recently Takada and collegues specifically investigate the effects of ppGpp / synthesis activity on the interaction of B. subtilis Rel. Please incorporate this paper into your discussion.

The article has now been referenced (line 325) (new reference 40).

12) Lines 312-303: ‘In conclusion, we present robust evidence demonstrating that the inactive HYD domain of RelA plays a novel regulatory role in controlling (p)ppGpp synthetase activity of the SYN domain.’ I am not sure that this is a novel conclusion. Clearly, the pseudo-HD is crucial for activity of RelA, given that it is conserved and retained though evolution. Mutations in this domain were shown earlier to abrogate the enzymatic activity (i.e. toxicity in ppGpp0 background, Montero et al. PLOS ONE 2014). Here the authors confirm these results but do not provide a mechanistic explanation.

We respectfully disagree with the reviewer and our argument for the novelty of this work is described above in a general response to reviewer 1.

13) Figure 1c: please use the structure from Tamman et al. (Rel complexed with native ppGpp) instead of the currently used (with pGpp).

We thank the reviewer for this suggestion. We have now substituted $_{Relseq}$ ^{for} Rel$_{Tte}$ (PDB:6S2T) reported in Tamman et al. as suggested by the reviewer.

Reviewer #2 (Remarks to the Author):

This manuscript describes is an important discovery regarding some very old questions. One is why the RelA protein E. coli and other Graham negative bacteria persist in evolution with a ppGpp synthetase but an inactive hydrolase but retains much of the sequence of an active domain. Most other bacteria have instead a bifunctional RSH gene with sequence homology with RelA. Both are able to self-regulate and balance an otherwise opposing activities to avoid an energy drain from a futile cycle of synthesizing (p)ppGpp and degrading it to GTP and GTP. How does RelA, a monofunctional (p)ppGpp synthetase regulates its synthesis activity without hydrolysis activity like most RSH proteins? The authors here describe the answer with a series of convincing experiments using RelA hypomorph mutants that define a 16-residue loop in the hydrolase-inactive domain that diminish ribosomal binding and manner independent of changing tRNA binding to ribosomes. Localization of effects of loop contacts on ribosomes is documented by an elegant sequencing methodology worked out earlier for mapping protein-RNA contacts by increased point mutants and deletions in rRNA. Alignments

of this region from many strains of this loop confirms its absence in RSH enzymes and presence in several Grahm negative RelA strains.

Critique
This work is clearly presented in a logical manner with convincing data. Publication is recommended. I have only minor problems with the manuscript.

We are grateful to the reviewer for appreciating our work and highlighting the importance of our work.

1) It is still puzzling to me that Δ116-129 (H-loop) increases SMG sensitivity & lowers (p)ppGpp accumulation (Fig 1) as if needed for synthesis activity whereas the quad mutants that neutralize charge and are plausibly argued to alter H-loop structure increases (p)ppGpp and SMG resistance above WT. On the surface this dilemma could be taken to argue the H-loop only provides a spacer function; interestingly the point mutants that lower ppGpp levels and SMG sensitivity argue against a simple spacer function.

We agree. Δ116-129 is a rather large modification to the enzyme and could have large effects on the overall structure of the protein, which could explain why the mutant is inactive. We now know that the H-loop is positioned between α6 and α7, which have been reported to move during the switching between OFF/ON (Tamman et al. 2020). We therefore believe that point mutants in this region, with lower activity are locked in a form with less flexibility that does not allow the switching mechanism to occur. This is consistent with the observation that we can introduce mutations that increase RelA activity (RelA^{QUAD}). Additionally, the loop is located in vicinity of the (p)ppGpp binding site of hydrolase active Rel proteins which is crucial for the switching mechanism.

2) There are two future experiments that might be interesting...just musing and not needed in this work to be published! One is to substitute the RelA H-loop for the missing H-loop in a well studied RelSeq RSH gene hydrolase/synthetase fragment lacking the C-terminal domain and see what happens. Also it might be interesting to turn the crank again and screen for mutants in a relA H-loop deletion with little synthetic activity and again screen for SMG-r mutants. Also screen for RelA quad mutants for slow growing suppressors.

We thank reviewer for suggesting these interesting experiments. If possible we will certainly do these experiments and incorporate the results in future piece of work.

3) Finally, Figure 4 makes it seem like the A121 residue is a very long way from the most mutagenic 2660 residue in the SRL domain.

We agree with this observation. The N-terminal domain is very flexible on the surface of the ribosome and could therefore more into vicinity of the SRL after binding to the ribosome. An example of that is the structures published in Loveland et al. 2016 in which the N-terminal domain of RelA moves closer to SRL depending on how tightly the tRNA is bound in the ribosomal A-site.

It is possible that the crosslinking with SRL occur transiently during binding to the ribosome. This has also been discussed in the manuscript (line 306-319)

4) Figures: 1. All have panel labels are placed so that they disappear when printed.

This should be fixed now.

5) Figs 1 & 2 label SMG resistance as “functional”. SMG resistance is more accurate.

We agree. We have changed the label for “SMG resistance” and have used this term throughout the manuscript.

6) 3-aminotriazole resistance on plates containing all AA-his provides another (less sensitive) measure of increased (p)ppGpp levels. The expectation is that weak ppGpp elevation would not be sensitive and high elevation would be resistant.

We agree and find this suggestion very interesting. We have now included M9 MM plates containing all AA-His, 1mM adenine , 1mMthiamine and with and without 15mM 3-amino-1,2,4-triazole as described in Gropp et al 2001 (Figure S1G). While the difference between un-tagged WT and RelA^{QUAD} was smaller using these plates the increased (p)ppGpp synthesis was still observed by increased growth within 12 hours of incubation. The tagged strain was very comparable to the SMG resistance assay (Figure S1F).

7) Line 132 inhibits should be singular... mutations inhibit.

Thank you very much. This has now been corrected.

Reviewer #3 (Remarks to the Author):

In the article by Sinha and Winther submitted to Communications Biology the authors investigate the regulatory role of a short loop in the tertiary structure of RelA, a conserved synthetase of guanosine penta and tetraphosphate (p)ppGpp, two stress response alarmones that cue physiological adaptation of bacteria. RelA is important for antimicrobial resistance and bacterial virulence gene regulation and pathogenesis. In general, bacteria have one enzyme, Rel/Spo, which acts as a both a synthetase and hydrolase of (p)ppGpp. Typically, the hydrolase domain (HYD) of the bifunctional enzyme is essential for bacterial growth and viability in the context of an intact synthetase and it has been shown that the hydrolase activity in the bifunctional enzyme is an important regulator of synthetase activity. For E. coli and most gamma proteobacteria, the hydrolase domain of RelA is degenerate and non-functional as a hydrolase of (p)ppGpp. These bacteria encode a second bifunctional enzyme
known as SpoT. The authors test the hypothesis that specific residues and secondary structures within the HYD of RelA are necessary for regulating synthetase activity of the enzyme in organisms that encode RelA and SpoT. They provide evidence in support of their prediction and use sequence alignments and a published crystal structure of the RelA-tRNA-ribosome complex to identify a short ~15 residue loop in the HYD, which is absent in hydrolase active Rel/Spo proteins. The authors employ cutting edge site-directed chromosomal mutagenesis of E. coli to generate a loop deletion mutant, and screen for substitutions within the loop that perturb RelA synthetase activity. They use assays of bacterial viability during amino acid starvation and biochemical measurements of (p)ppGpp to show that the loop and particular residues within the loop are necessary for promoting the synthetase activity of RelA. They expand their observations by using chemical crosslinking and mass spectrometry to
probe the effects of the loop mutants on complex formation. They define differences in the degree of interaction between RelA and the ribosome that might explain the change in synthetase

activity conveyed by perturbing the regulatory loop in the HYD.

This is a highly rigorous examination of the role of the HYD loop in the regulation of RelA, a critical protein for bacteria. The work is comprehensive and focused. It is fully appropriate for the mission and scope of this journal and should be broadly read. The use of site directed substitution mutagenesis and genetic screening to define structure function principals is exemplary. Also, the chemical crosslinking and mass spectrometry to define differences in interactions within the RelA-ribosome complex was impressive, especially since they included 5 min and 30 min post starvation time points and measured the interactions in bacteria cells expressing wild type and mutant proteins. I recommend that the work be accepted with minor revisions. I offer only minor critiques and criticisms that might make the work more coherent and comprehendible for naïve audiences.

We are grateful to the reviewer for the positive and encouraging words.

Semi Major Critique.

1) Other than western blots, which were hard to interpret, there is no measurement of protein folding for the tagged RelA variants. Something like, circular dichroism or tryptophan fluorescence could be conducted. Perhaps the cross-linking somehow shows this already. If so, than it should be stated explicitly.

We thank the reviewer for pointing out this concern. As suggested by the reviewer the cross linking patterns (Figure 4) clearly suggest that $RelA^{A121E}$ is structurally stable and binds to the ribosome similar to the wild type protein. We have pointed this out in the manuscript (lines 299-302).

Minor Critiques

2) Line 39 Are ppGpp and pppGpp truly “analogs” of GDP/GTP? Perhaps a different adjective is needed.

We agree. The sentence now reads “Derivatives of GDP/GTP, guanosine tetra- and pentaphosphate (collectively referred to as (p)ppGpp or alarmones), are the effector molecules of the stringent response and are synthesized/hydrolyzed by the RSH superfamily (RelA/SpoT homologues) proteins.” (line 39)

3) Line 54 “hungry” is not an appropriate term for a ribosome. Did you mean “apo” in line 65 empty is used, this might be better?

We agree. The sentence now reads: “RelA activation occurs when RelA binds with an uncharged tRNA at an empty A-site of a stalled ribosome, which leads to induction of (p)ppGpp synthesis” (line 54-55).

4) Line 103 SMG plates is not appropriately introduced or discussed as a RelA activity assay. Please explain how this biological assay works early in the results.

High concentrations of single carbon amino acids serine, methionine and glycine induce isoleucine starvation as reported by Uzan and Danchin 1978. A sentence which specifies this has been included at line 104-106:” RelA is essential for growth on SMG plates, which contain high

concentrations of single carbon amino acids serine, methionine and glycine and leads to isoleucine starvation".

5) Lines 106-108 Not sure why the specific results of this study are stated, I suggest removing these sentences and replace with a description of SMG plates

This study was included as it previously identified substitutions in RelA that affected RelA activity and allowed deletion of the otherwise essential spot gene. This study also identified four mutations in the pseudo hydrolase domain. Two of these mutations were in or in the vicinity of the H-loop identified here.

6) Line 114 What is Relseq?

Relseq is the Rel protein of *streptococcus dysgalactiae* . This has now been changed to RelTte of Thermus thermophilus as advised by reviewer 1. The structure of the hydrolase domain of RelTte is compared with pseudo hydrolase domain of RelA in Figure 1C.

7) The acronyms become really brutal for a naïve reader, is HTF necessary in the main text. Is this the tag? Can it be removed or simplified?

We agree. But as the study also includes un-tagged versions as control we feel the need of including HTF in the description. To clarify this we have now moved the description of the HTF tag to the beginning of the results section (see line106-108).

8) Line 538 The title of this figure does not read well. Perhaps something like "Residues of 114-130 of the RelA pseudo-hydrolase domain form a loop that controls ppGpp synthesis"

We agree and thank the reviewer for the suggestion. The title (line 621) has been changed accordingly.

9) Line 558 To clarify, can you briefly explain why L-Valine prompts isoleucine starvation?

Three isozymes (ilvGM, ilvBN and ilvIH) are shared between the valine and isoleucine biosynthetic pathways. In the canonical *Escherichia coli* K-12 wild type strain there is a frameshift mutation in the ilvGM gene and the two other isozymes ilvBN and ilvIH are susceptible to feed-back inhibition by L-Valine. As a consequence *E.coli* K-12 becomes auxotrophic for isoleucine at high concentrations of L-Valine.

We have included reference 42 (Leavitt and Umbarger 1962) in materials and method (line 344). This study initially reported this phenomenon.

0) Line 570-571 Please cite the Salmonella PLOS-One paper that was the first to use this method in the main text and in this legend?

We have included the reference in the figure legend (line 651).

REVIEWERS' COMMENTS:

Reviewer #3 (Remarks to the Author):

I appreciate the authors diligent work to revise the manuscript. Each of my concerns were appropriately addressed and I move for acceptance of the document.

Response to referees' comments, 12th of February 2021

COMSSBIO-20-2293B

Please find below a response (in blue) to the referee.

REVIEWERS' COMMENTS:

Reviewer #3 (Remarks to the Author):

I appreciate the authors diligent work to revise the manuscript. Each of my concerns were appropriately addressed and I move for acceptance of the document.

We thank the referee for the appreciation of our work and the positive review.
